# A novel, dual pan-PIM/FLT3 inhibitor SEL24 exhibits broad therapeutic potential in acute myeloid leukemia

**DOI:** 10.18632/oncotarget.24747

**Published:** 2018-03-30

**Authors:** Wojciech Czardybon, Renata Windak, Aniela Gołas, Michał Gałęzowski, Aleksandra Sabiniarz, Izabela Dolata, Magdalena Salwińska, Paweł Guzik, Magdalena Zawadzka, Ewelina Gabor-Worwa, Bożena Winnik, Małgorzata Żurawska, Ewa Kolasińska, Ewelina Wincza, Marta Bugaj, Monika Danielewicz, Eliza Majewska, Milena Mazan, Grzegorz Dubin, Monika Noyszewska-Kania, Ewa Jabłońska, Maciej Szydłowski, Tomasz Sewastianik, Bartosz Puła, Anna Szumera-Ciećkiewicz, Monika Prochorec-Sobieszek, Elżbieta Mądro, Ewa Lech-Marańda, Krzysztof Warzocha, Jerome Tamburini, Przemysław Juszczyński, Krzysztof Brzózka

**Affiliations:** ^1^ Selvita S.A., Krakow, Poland; ^2^ Malopolska Centre of Biotechnology, Krakow, Poland; ^3^ Dept. of Experimental Hematology, Institute of Hematology and Transfusion Medicine, Warsaw, Poland; ^4^ Dept. of Diagnostic Hematology, Institute of Hematology and Transfusion Medicine, Warsaw, Poland; ^5^ Dept. of Hematology, Institute of Hematology and Transfusion Medicine, Warsaw, Poland; ^6^ Dept. of Hematology and Transfusion Medicine, Centre of Postgraduate Medical Education, Marymoncka, Warsaw, Poland; ^7^ Institut Cochin, Département Développement, Reproduction, Cancer, Paris, France; ^8^ Faculté de Médecine, Université Paris Descartes, Sorbonne Paris Cité, Paris, France; ^9^ Equipe Labellisée, Ligue Nationale Contre le Cancer (LNCC), Paris, France

**Keywords:** PIM kinase, FLT3 kinase, dual inhibitor, targeted therapy, AML

## Abstract

Fms-like tyrosine kinase 3 internal tandem duplication (FLT3-ITD) is one of the most common genetic lesions in acute myeloid leukemia patients (AML). Although FLT3 tyrosine kinase inhibitors initially exhibit clinical activity, resistance to treatment inevitably occurs within months. PIM kinases are thought to be major drivers of the resistance phenotype and their inhibition in relapsed samples restores cell sensitivity to FLT3 inhibitors. Thus, simultaneous PIM and FLT3 inhibition represents a promising strategy in AML therapy. For such reasons, we have developed SEL24-B489 - a potent, dual PIM and FLT3-ITD inhibitor. SEL24-B489 exhibited significantly broader on-target activity in AML cell lines and primary AML blasts than selective FLT3-ITD or PIM inhibitors. SEL24-B489 also demonstrated marked activity in cells bearing FLT3 tyrosine kinase domain (TKD) mutations that lead to FLT3 inhibitor resistance. Moreover, SEL24-B489 inhibited the growth of a broad panel of AML cell lines in xenograft models with a clear pharmacodynamic-pharmacokinetic relationship. Taken together, our data highlight the unique dual activity of the SEL24-B489 that abrogates the activity of signaling circuits involved in proliferation, inhibition of apoptosis and protein translation/metabolism. These results underscore the therapeutic potential of the dual PIM/FLT3-ITD inhibitor for the treatment of AML.

## INTRODUCTION

Acute myeloid leukemia (AML) is a clonal disease of hematopoietic progenitor cells that are unable to differentiate [1]. The current standard of care, based on chemotherapy and allogeneic hematopoietic stem cell transplantation, results in an overall cure rate of about 40% [2, 3]. AML is a heterogeneous disease, harboring numerous cytogenetic and/or submicroscopic lesions that determine disease aggressiveness. Some of these lesions have been credentialed as potential therapeutic targets, but despite these discoveries, little progress in clinical outcome has been achieved in AML patients over the last decade. One of the first molecular abnormalities described in AML were the fms-like tyrosine kinase 3 (FLT3) gene mutations. FLT3 is a type 3 receptor tyrosine kinase, which in non-pathological conditions is expressed on hematopoietic stem cells, but lost upon their maturation [4, 5]. Approximately 70% of newly diagnosed AML cases are characterized by expression of FLT3, and ~30% of patients exhibit activating mutations of the receptor (i.e. internal tandem duplications in the juxtamembrane domain (FLT3-ITD) found in approximately 25% patients and point mutations in the activation loop of the tyrosine kinase domain (FLT3-TKD) in an additional 7% of cases) [6–8]. Mutated FLT3 phosphorylates several key signaling molecules such as signal transducer and activator of transcription 5 (STAT5), mitogen activated protein kinase (MAPK) and BCL2-associated death promoter (BAD), fostering proliferation and survival of AML blasts [9–13]. The FLT3-ITD mutations are adverse prognostic factors associated with increased blast counts in peripheral blood and bone marrow, short remission durations and high relapse rates [6, 14–16]. Although up to 70% of AML patients showed hematological improvement in clinical trials with FLT3 inhibitors, the responses were transient and inevitably followed by the disease progression [17]. The failure of therapy with FLT3 inhibitors is thought to be at least in part caused by a selection of AML clones harboring various TKD mutations and activation of downstream effector pathways, including constitutive STAT5 activity [18–21]. In a recent study, relapse samples from AML patients treated with FLT3 inhibitors demonstrated increased expression of PIM kinases. Importantly, in AML bearing FLT3-ITD mutations, PIM1 and PIM2 were identified as STAT5 downstream targets and mediators of increased blast survival and clonogenic potential [9, 11]. Consistent with this, ectopic PIM2 expression induced resistance to FLT3 inhibition in both FLT3-ITD*–*induced myeloproliferative neoplasm and AML models in mice [22], underscoring the role of PIMs in the emergence of this phenotype.

PIM kinases (proviral integration site for Moloney murine leukemia virus), comprising three highly homologous serine/threonine kinases, are involved in pathogenesis and progression of multiple malignancies, including AML [11, 23–29]. PIM kinases are major oncogenes, regulating several important cellular processes, such as the cell cycle, differentiation, proliferation, apoptosis, migration, translation, transcription and membrane transport [9, 11, 30–32]. Unlike most kinases, PIMs do not possess a regulatory domain and once expressed, they become constitutively active [33]. Mice deficient in all PIM kinases are characterized by reduced body weight and impaired responses to hematopoietic growth factors, but they remain viable and fertile, suggesting that PIMs could be safely therapeutically targeted [34]. Although in earlier studies with preclinical AML models, a small molecule SGI-1776 PIM/FLT3 inhibitor exhibited promising activity, the compound failed clinical testing due to a narrow therapeutic window and cardiac toxicity (related to off-target effects of the inhibitor and its metabolites - inhibition of the potassium channel hERG) [25, 35]. Newer, potentially more specific compounds targeting PIM kinases (LGH447 and INCB053914) are currently under clinical development in hematological malignancies.

Since PIM kinases have emerged as important effectors and mediators of FLT3-ITD activity, we hypothesized that dual inhibition of FLT3 and PIM kinases could lead to improved efficacy and be a promising approach in overcoming the rapid development of resistance to such agents [25, 36–38]. We have recently developed first-in-class, dual PIM/FLT3 kinase inhibitor, the SEL24-B489 compound, and profiled its activity in *in vitro* and *in vivo* AML models [39]. Herein, we show that SEL24-B489 specifically inhibits PIM- and FLT3-ITD- related pathways and exhibits significantly broader anti-tumor activity in AML models than selective FLT3-ITD or PIM inhibitors, underscoring its therapeutic potential for the treatment of AML.

## RESULTS

### SEL24-B489 is a potent PIM/FLT3-ITD inhibitor with antiproliferative activity against AML cell lines

SEL24-B489, 5,6-dibromo-4-nitro-2-(piperidin-4-yl)-1-(propan-2-yl)-1H-1,3-benzodiazole (Supplementary Figure 1) is a potent, type I, dual PIM/FLT3 inhibitor, with a Kd of 2 nM for PIM1, 2 nM for PIM2 and 3 nM for PIM3 (Table 1). Comparison of Kd for wild type FLT3 (FLT3-WT) with internal tandem duplication mutated FLT3 (FLT3-ITD) revealed a 10-fold stronger inhibitor binding in the latter (160 nM vs 16 nM for FLT3-WT and FLT3-ITD, respectively).

**Table 1:** SEL24-B489 kinase inhibition profile

	Kd [nM]		
Kinase	SEL24-B489	AZD1208	AC220
**PIM1**	2	0.2	-
**PIM2**	2	0.8	-
**PIM3**	3	0.9	-
**FLT3wt**	160	-	1
**FLT3 (ITD)**	16	-	9
FLT3 (D835H)*	37	-	-
FLT3 (D835V)*	11	-	-
FLT3 (D835Y)*	16	-	-
FLT3 (ITD)*	12	-	-
FLT3 (ITD, D835V)*	2.8	-	-
FLT3 (ITD, F691L)*	10	-	-
FLT3 (K663Q)*	530	-	-
FLT3 (N841I)*	150	-	-
FLT3 (R834Q)*	3200	-	-

Dissociation constants (Kd) were measured at DiscoverX. (^*^) indicates specific screen against FLT3 mutants.

**Figure 1:**
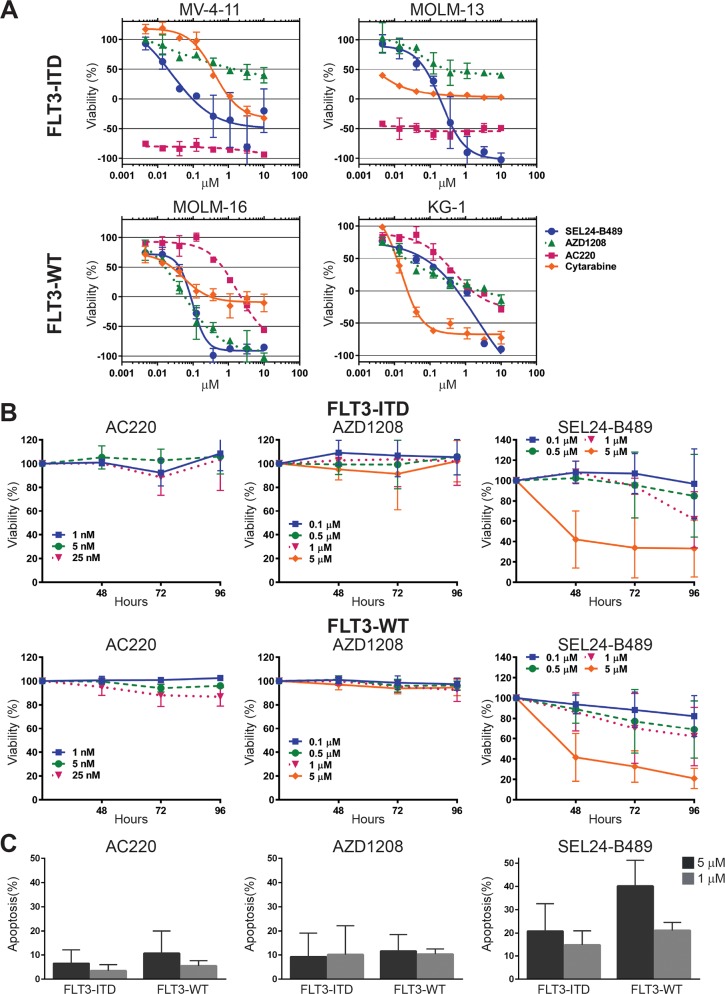
*In vitro* activity of SEL24-B489 in AML cell lines and primary AML blasts **(A)** *In vitro* activity of SEL24-B489, AZD1208, AC220 and AraC in AML cell lines (FLT3-ITD+ and FLT3-WT) was assessed using an MTS assay. Cells viability was measured after 72 h incubation with three-fold serial dilutions of compounds, starting at 10 μM concentration. Obtained data were presented as percentage of viable cells compared with control (untreated) cells viability. Error bars indicate SD. **(B)** *In vitro* activity of SEL24-B489, AZD1208, AC220 in primary AML blasts obtained from peripheral blood of three FLT3-ITD+ and three FLT3-WT patients was assessed individually with the MTS assay at three indicated time points. Error bars indicate SD. **(C)** *In vitro* activity of SEL24-B489, AZD1208, AC220 in primary AML blasts obtained from bone marrow of 13 AML patients was assessed with the MTS assay at two indicated concentrations. Error bars indicate SD.

Since SEL24-B489 inhibits FLT3 mutants and simultaneously blocks activity of PIM kinases (the key downstream effectors of FLT3 signaling), we hypothesized that these properties should lead to broader toxicity of the compound against AML cells in comparison to selective PIM or FLT3 inhibitors. For this reason, we compared SEL24-B489 head-to-head with a selective PIM inhibitor (AZD1208) and a selective FLT3-ITD inhibitor (AC220) in a panel of AML cell lines with FLT3-ITD (MV-4-11, MOLM-13) or unmutated kinase (FLT3-WT: KG-1, MOLM-16). SEL24-B489 exhibited a significantly broader activity, irrespective of FLT3 status, than either of the selective inhibitors (Figure 1A, Supplementary Figure 5A, Supplementary Table 3 and 4). Activity of SEL24-B489, depending on the driving oncogene mutation, could be a result of activity on PIM, FLT3-WT, FLT3 mutants or an effect of synergy in inhibition of both PIM and FLT3 (WT or mutants). AC220 was selectively active against FLT3-ITD+ cells (MV-4-11, MOLM-13), but exhibited only a minor effect in cells with FLT3-WT (MOLM-16, KG-1). In agreement with earlier studies [40], the selective PIM inhibitor AZD1208 exhibited strong toxicity against MOLM-16 cells (FLT3-WT, high PIM expression), but was less active against FLT3-mutated cell lines (Figure 1A, Supplementary Table 3 and 4). To determine the mechanism of response to SEL24-B489, we performed multiparametric cell cycle analysis in FLT3-ITD+ (MV-4-11 and MOLM-13) and FLT3-WT (KG-1) cell lines. In MOLM-13 and to a lesser extent in MV4-11 cells, we observed a dose-dependent disruption of cell cycle with especially pronounced depletion of the S phase after treatment with SEL24-B489, accompanied by PARP cleavage and apoptosis (Supplementary Figures 2 and 3). As expected, AC220 also induced apoptosis and cell cycle arrest, whereas the selective PIM kinase inhibitor LGH447 had a much weaker effect. For the KG-1 cell line, we also observed 2-fold induction of PARP cleavage (Supplementary Figure 4) although the overall effect was weaker for all compounds.

Consistent with these findings, SEL24-B489 was more active against primary FLT3-WT and FLT3-ITD peripheral AML cells and CD34+ bone marrow blasts than either of the selective inhibitors (Figure 1B and 1C, respectively).

To further corroborate the advantage of dual PIM/FLT3-ITD inhibition, we simultaneously inhibited PIMs and FLT3-ITD using a combination of the selective pan-PIM inhibitor AZD1208 and the selective FLT3 inhibitor AC220 in a FLT3-ITD dependent cell line MV-4-11. As expected, the combination did exhibit a synergistic effect (Supplementary Figure 5B). SEL24-B489 also showed a marked synergy with AraC or vosaroxin in a broad range of concentrations in the same FLT3-ITD dependent cell line MV-4-11. Overall, these results highlight the therapeutic potential of a SEL24-B489 inhibitor with dual specificity against FLT3 and PIM.

### Activity of SEL24-B489 in cells with FLT3 TKD mutations associated with resistance to FLT3 inhibitors

**Figure 2:**
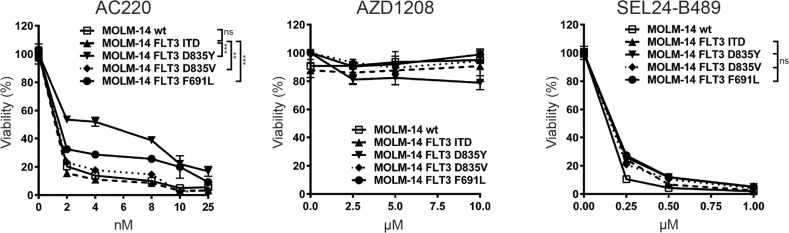
SEL24-B489 decreases viability of AML cells with FLT3-TKD mutations associated with resistance to selective FLT3-ITD inhibitors Parental MOLM-14 cells and cells transduced with either FLT3-ITD, FLT3 D835Y, FLT3 D835V or FLT3-F691L were incubated with indicated compounds for 72 h and cellular viability was assessed by the MTS assay. Differences in responses of cells bearing FLT3-TKD mutants to AC220, a FLT3-ITD selective inhibitor, are indicated with asterisks (^***^ for p<0.0001, ^**^ for p<.001, factorial ANOVA with Tukey's post-hoc test). SEL24-B489 demonstrated activity against all mutants (p=ns). Error bars indicate SD.

Since PIM kinases have emerged as important mediators of FLT3-inhibitor resistance [22], we hypothesized that the dual specificity of SEL24-B489 against FLT3-ITD and PIMs might overcome the phenotype of resistance. We tested the inhibitory properties of SEL24-B489 against different FLT3 mutants (Table 1). SEL24-B489 showed strong binding to FLT3 mutant kinases at low nM Kd values for 6 out of 9 tested mutants (Table 1). To further test this hypothesis, we utilized previously developed MOLM-14 cells transduced with either FLT3-WT or FLT3 alleles containing TKD point mutations (D835Y, D835V and F691L). In these experiments, we compared head-to-head activity of SEL24-B489 with AZD1208 and AC220. The AZD1208 PIM inhibitor only marginally affected the viability of tested cell lines regardless of their FLT3 status. The cells expressing resistance-associated FLT3-TKD mutants exhibited lower sensitivity to AC220 than cells transduced with FLT3-ITD or parental MOLM-14 cells. In marked contrast, neither of the FLT3 TKD point mutations decreased the cellular sensitivity to the dual FLT3-ITD/PIM inhibitor SEL24-B489 (Figure 2), underscoring the role of PIM kinases as key FLT3-signaling effectors and circumstantiating the concept of dual PIM-FLT3-ITD inhibition in AML targeted therapy.

### Biochemical consequences of selective versus simultaneous inhibition of FLT3-ITD and PIM kinases

**Figure 3:**
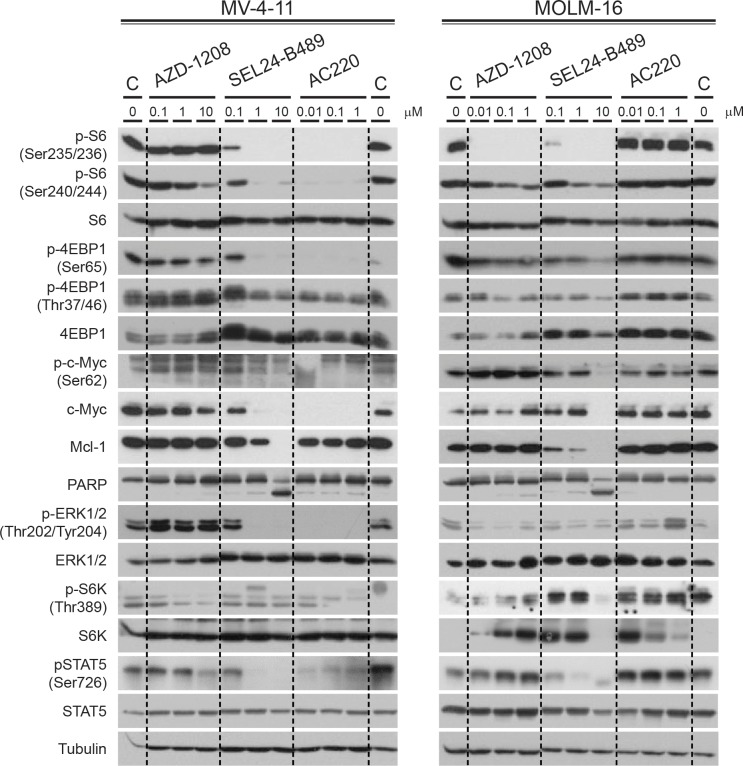
Effect of SEL24-B489 treatment on FLT3 and PIM kinase downstream signaling in AML cell lines MV-4-11 (FLT3-ITD+) and MOLM-16 (FLT3-WT) cells, both expressing high levels of PIM kinases, were treated with SEL24-B489 for 4 hours in a dose-dependent manner and analyzed for phosphorylation of FLT3/PIM kinase downstream targets using Western blot. AZD1208 and AC220 compounds were used as reference. C – vehicle control.

To confirm the on-target activity of SEL24-B489 inhibitor and to compare the consequences of selective versus simultaneous inhibition of FLT3-ITD and PIM kinases *in vitro*, we used MV-4-11 (FLT3-ITD+) and MOLM-16 (FLT3-WT) cells and treated them with increasing concentrations of SEL24-B489, AC220 or AZD1208 for 4 hours. Thereafter, we assessed the activities of FLT3-ITD – induced key pro-survival signaling pathways; STAT5, RAF/MEK/ERK and PI3K/AKT (Figure 3). We also assessed the activity and abundance of PIM kinase substrates: pS6 (S^235/236^), p4EBP1 (S^65^) and c-MYC (Figure 3). In MV-4-11 cells, selective FLT3-ITD inhibitor AC220 and dual FLT3-ITD/PIM inhibitor SEL24-B489 completely blocked STAT5 signaling, RAS/RAF/MEK activity (measured by decreased pERK1/2 Y^202/204^ levels) and PI3K/mTOR signaling (measured by decreased phosphorylations of p70S6K T^389^, pS6 S^240/244^, and p4EBP1 T^37/46^). FLT3-ITD and dual FLT3-ITD/PIM inhibitors also decreased c-MYC abundance, at least in part due to decreased c-MYC S^62^ phosphorylation. Since the expression of a major anti-apoptotic BCL2 family member, MCL1, is regulated by translation, and PIM kinases regulate protein synthesis through 4EBP1, we also assessed the abundance of MCL1. Expression of this protein markedly decreased upon SEL24-B489 treatment and - to a lesser extent - upon AC220. Consistent with this, we noted a marked induction of apoptosis, indicated by PARP cleavage, in cells treated with SEL24-B489, but not with other tested inhibitors. In FLT3-ITD positive MV-4-11 cells, the selective pan-PIM inhibitor AZD1208 led to a moderate decrease in PIM substrates activity, pS6 and p4EBP1, but did not change the STAT5, RAF/ERK1/2 and AKT signaling and did not alter c-MYC and MCL1 abundance.

In the FLT3-WT cell line MOLM-16, FLT-ITD-specific inhibitor AC220, did not alter activity of assessed pathways and abundance of c-MYC and MCL1. PIM inhibitor AZD1208 and SEL24-B489 caused a profound inhibition of S6 (S^235/236^), but had little effect on PI3K/mTOR signaling. Moderate inhibition of p70S6K (Thr^389^) after incubation with SEL24-B489 and AZD1208 are most likely caused by an indirect effect of PIM inhibition on mTOR signaling [41–43]. Within the on-target range of concentrations (0.1-1 μM), only SEL24-B489 inhibited STAT5 (Ser^726^) and reduced expression of MCL1, whereas none of the selective inhibitors altered c-MYC abundance or induced PARP cleavage. In line with our finding Maria Baer's group also reported that concurrent inhibition of PIM and FLT3 post-translationally downregulates the anti-apoptotic protein MCL1 through downregulation of the USP9X deubiquitinase [44]. Given the profound and robust decrease in pS6 (S^235/236)^ abundance in AML cells after PIM inhibition, we hypothesized that this parameter might be utilized to monitor SEL24-B489 pharmacodynamics in future clinical trials. Thus, we developed a quantitative FACS-based assay to evaluate changes in S6 (S^356/236^) phosphorylation levels. Both FACS assay and Western blotting showed dose-dependent downregulation of pS6 (Supplementary Figure 6), what was further confirmed by highly concordant IC50 values for both methods (Supplementary Table 5).

Taken together, these data indicate that dual FLT3-ITD/PIM inhibitor elicits broader changes in pro-survival intracellular signaling than the specific PIM or FLT3 inhibitors alone, and that these robust changes might be utilized as clinically relevant biomarker.

### SEL24-B489 exhibits activity in AML *in vivo* models

**Figure 4:**
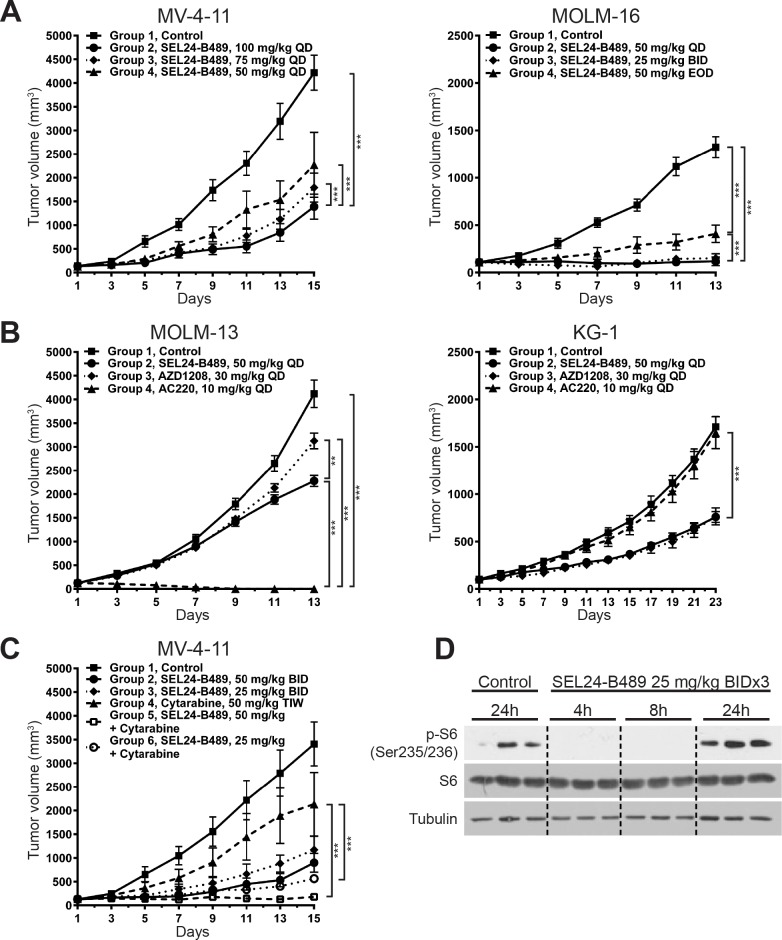
SEL24-B489 inhibits growth of AML xenografts **(A-C)** MOLM-16, MV-4-11, KG-1 and MOLM-13 cell lines were implanted subcutaneously in immunodeficient mice (SCID/beige for MOLM-16 and MV-4-11; SCID for KG-1 and MOLM-13). When tumors reached 100 to 130 mm^3^, mice were treated with SEL-B489, AC220, AZD1208, AraC or with indicated combinations. Tumor growth was monitored every other day for up to 23 consecutive days. Error bars indicate SEM. Differences in tumor growth kinetics were calculated using factorial ANOVA with Tukey's post hoc test (^***^ for p<0.0001; ^**^ for p<0.001). **(D)** PK/PD relationship between SEL24-B489 and S6 (S^235/236^) expression in tumors explanted from inhibitor treated mice. SEL24-B489 was given by oral gavage BID (25 mg/kg) for three consecutive days; after the last dose, the animals were sacrificed at indicated time points and tumor samples were harvested for biomarker response analysis.

After demonstrating the activity of SEL24-B489 against FLT3-WT and FLT3-ITD AML cell lines, we sought to determine the *in vivo* activity of our inhibitor. First, we determined the compound's PK and metabolic stability in mice, rats and dogs (Supplementary Tables 6–8). Having confirmed favorable pharmacokinetics, good bioavailability and metabolic stability of the compound, we tested SEL24-B489 single – agent activity in animal xenograft models. In SCID/beige mice bearing MV-4-11 tumors (FLT3-ITD+) treated with SEL24-B489, we noted marked dose – dependent tumor reduction (67%, 74% and 82% tumor growth inhibition (TGI) for 50, 75 and 100 mg/kg daily doses, respectively) (Figure 4A). Even more profound responses were found in MOLM-16 (FLT3-WT) tumor-bearing SCID/beige mice: ~100% TGI was reached at doses 50 mg/kg in QD schedule and 25 mg/kg in BID schedule (Figure 4A). Dosing of 50 mg/kg in EOD schedule resulted in 79% TGI.

Next, to further confirm SEL24-B489 activity *in vivo* and compare it to selective PIM (AZD1208) and FLT3 (AC220) inhibitors, we established MOLM-13 and KG-1 xenografts in SCID mice. AC220 (10 mg/kg, QD) resulted in 100% TGI in the FLT3-ITD positive MOLM-13 cell line (Figure 4B). In marked contrast, for the KG-1 (FLT3-WT) tumor bearing animals, AC220 treatment did not significantly inhibit tumor cell growth (TGI=5.4%). PIM inhibitor AZD1208 had a different activity in those two models of 19.5% and 59.9% TGI in MOLM-13 and KG-1, respectively. Comparable results were noted in SEL24-B489-treated animals (~50% TGI in both types of tumors) (Figure 4B). We can speculate that the apparent lower activity of SEL24 vs AC220 *in vivo* can be explained by relatively short half-life of SEL24 *in vivo* in mouse, and we observed higher TGI in xenograft studies after BID dosing of SEL24 (data not shown).

Given the marked synergy between SEL24-B489 and AraC observed *in vitro*, we assessed the combined effects of these two drugs in MV-4-11 xenograft models. SEL24-B489 was tested at two doses, 50 and 25 mg/kg, administered twice daily alone or in combination with AraC administered at a 50 mg/kg dose three times weekly. In line with the previous experiments, SEL24-B489 exhibited dose-dependent activity (Figure 4C). AraC alone decreased tumor growth by 66%. Consistent with the *in vitro* data indicating marked synergy between these drugs, a combination of AraC with 25 mg/kg BID of SEL24-B489 resulted in 89% TGI, whereas when combined with 50 mg/kg of SEL24-B489 it almost completely blocked tumor growth *in vivo* (99% TGI). Of note, after 14 days of consecutive administration of either drug alone or in combination, hematological and biochemical parameters were not different from those in the control mice (Supplementary Tables 9–10).

Because subcutaneous AML xenografts in mice lack the translation into a systemic disease and do not capture the complexity of AML progression, we decided to further confirm preclinical efficacy of SEL24-B489 by investigating the PK/PD relationship that is the modulation of PIM substrate activity in a time course fashion. SCID/beige mice xenografted subcutaneously with MOLM-16 tumors were treated with SEL24-B489 (25 mg/kg) inhibitor for 3 consecutive days in a BID schedule and after the last administration, animals were sacrificed at indicated time points and tumor samples were then harvested for PK/PD response analysis (Figure 4D). Phospho-S6 remained suppressed up to 8 hours after the last dose of SEL24-B489, indicating that the observed biological effects are consistent with target engagement at the tumor site.

## DISCUSSION

**Figure 5:**
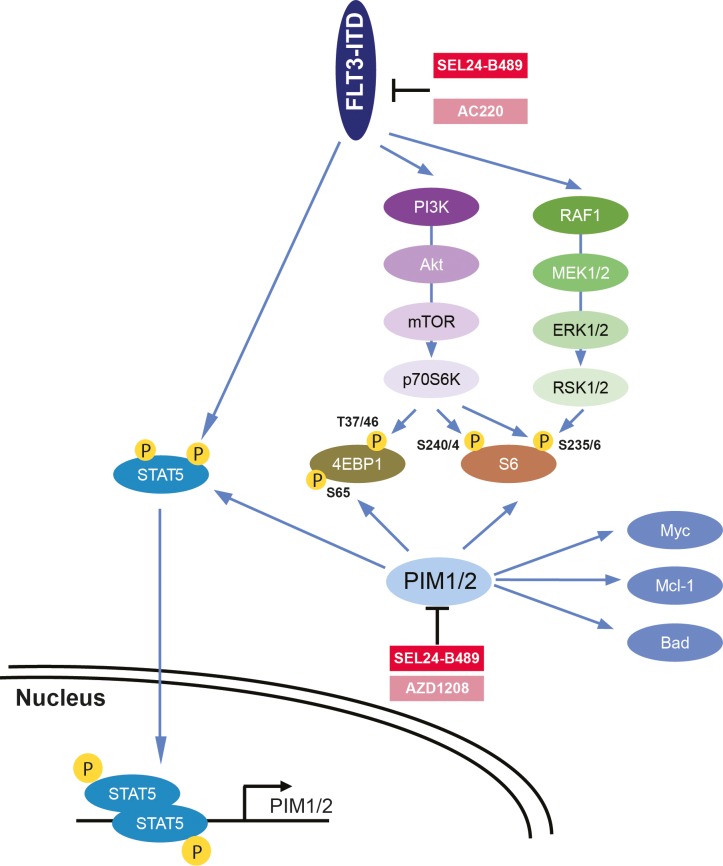
Diagram summarizing key biochemical consequences of selective versus combined inhibition of FLT3-ITD and PIM kinases

In this study, we have characterized the activity of a newly developed, dual inhibitor of PIM kinases and FLT3-ITD, SEL24-B489, in pre-clinical models of AML. The inhibitor exhibited on-target activity at sub/low-micromolar concentrations and efficiently blocked both FLT3-ITD-triggered pathways and phosphorylation of PIM substrates, attenuating survival of AML cell lines and primary AML blasts. Since PIM kinases emerge as important mediators of FLT3-induced signaling, these findings highlight the potential clinical benefits of dual inhibitor activity (Figure 5).

FLT3–ITD mutations are among the most common genetic lesions in AML and are associated with inferior prognosis, circumstantiating the development of FLT3's inhibitors. FLT3-ITD knock-ins drive leukemogenesis from early hematopoietic progenitor cells by activating its direct substrates - including STAT5, MAPK/ERK and PI3K/AKT pathways that lead to block of cellular differentiation and acceleration of cell growth, metabolism, proliferation, along with inhibiting apoptosis [45–47]. FLT3 mutations and signaling are also thought to play a critical role in the survival of leukemic stem cells via activation of STAT5 dependent transcription [12, 48, 49].

In AML blasts, FLT3-induced STAT5 activity triggers expression of PIM1 and PIM2 kinases [22, 50]. PIMs are thus a downstream target of FLT3 signaling, consolidating and amplifying its oncogenic potential [22, 23, 25, 50]. Furthermore, once expressed, PIMs in AML cells are known to stabilize additional oncogenic circuits, such as c-MYC, and support cell migration and homing by increasing surface expression of CXCR4 [32, 51–53]. In addition, forced overexpression of PIMs in AML cells decreases sensitivity of AML blasts to targeted FLT3-ITD inhibition by a mechanism that likely involves a direct phosphorylation of FLT3 by PIMs, reducing affinity of the FLT3-ITD receptor to its inhibitors [22, 50]. As a consequence, inhibition of PIM kinases restores sensitivity to FLT3 inhibitors in this setting. Thus, since drug resistance invariably occurs in AML patients treated with FLT3-ITD inhibitors, targeting the axis proximally (FLT3-ITD) and distally (PIMs) is thereby a rational strategy that might circumvent the development of resistance (Figure 5).

Although PIM kinases share certain substrates and exhibit partial functional redundancy, they also possess isoform–specific functions that are particularly important for tumor biology. For example, PIM1 regulates cell migration, homing and apoptosis by modulating CXCR4 and BAD phosphorylation, respectively, whereas PIM2 is involved in translational control by phosphorylating 4E-BP1 [32, 54, 55]. Thus, simultaneous inhibition of all three PIM isoforms will exhibit broader biological consequences than targeting single isoform, as such approach will preclude potential functional compensation due to isoform redundancy, but also eliminate isoform-unique oncogenic activity [24, 53].

Consistent with the functional dependencies linking FLT3 signaling, PIM kinases and FLT3-ITD inhibitor resistance, our newly developed, first-in-class SEL24-B489 inhibitor blocked the activities of direct FT3-ITD substrates (STAT5, MAPK/ERK) and simultaneously inhibited phosphorylation of key PIM substrates (4EBP1, S6, c-MYC). In contrast, selective pan-PIM (AZD1208) or FLT3-ITD (AC220) inhibitors exhibited narrow on-target activities. Consistent with this finding, we noted superior and broader activity of the SEL24-B489 than with either of the inhibitors in different cell line xenograft models. Furthermore, SEL24-B489 was toxic to FLT3-ITD transduced cells bearing resistance-inducing TKD mutations, confirming the role of PIMs in driving the FLT3-ITD inhibitor resistance. Thus, targeting the functional FLT3-ITD axis proximally and distally might be a clinically attractive strategy.

Of note, PIM kinases are expressed and exhibit catalytic activity in also FLT3-WT AML cells [56]. In such cases, their expression is likely driven by activated STATs induced by other activated oncogenic kinases, or other myeloid transcription factors, such as HOXA9 [57]. Regardless of the underlying mechanisms inducing expression of PIMs in these FLT3-WT AMLs, we show that their inhibition triggers cell death *in vitro* and tumor growth inhibition *in vivo* with similar efficacy as in cells bearing FLT3-ITD mutations. These results are consistent with other studies indicating that PIM kinase inhibition is cytotoxic also in FLT3-WT AMLs, in a mechanism involving attenuation of STAT5 activity and destabilization of MYC [53]. Thus, the “PIM component” of the dual inhibitor SEL24-B489 prompts speculations that the compound will also be clinically active in patients without FLT3-ITD, but with PIM kinase expression and addiction. From the clinical standpoint, it is crucial to identify potential responders using knowledge-based, biomarker-driven criteria. For FLT3-ITD mutations the “binary” biomarker is clearly available, but for FLT3-WT-tumors, it is less apparent. Herein, we observed robust inhibition of pS6 level in all SEL24-B489-sensitive cell lines, including those with FLT3-WT. Thus, changes in pS6 levels might be used in clinical trials as an early surrogate indicator of inhibitor's activity. For this purpose, we developed a FACS-based assay, which produced quantitative, robust results, highly concordant with Western blot analysis.

Recent data indicate that AML blasts with surface expression of a leukemic stem cell and a conspicuous prognostic marker CD25 are more sensitive to PIM inhibition. Since CD25 is a STAT5 regulated gene, it might be a mechanistically relevant, predictive biomarker for sensitivity to PIM kinase inhibitors. Inhibition of PIMs in CD25+ AML cells attenuated STAT5 activation and destabilized c-MYC, indicating that PIMs are involved in molecular circuitry supporting leukemic stem cells [53]. For such reasons, inhibition of PIMs in AML patients might lead to elimination of leukemia-initiating cells, the major source of relapse and treatment failures. Thus, targeting FLT3-PIM axis in AML is a promising, rational strategy to deplete these tumor-driving signals, increase the cytotoxicity of conventional chemotherapeutics, and improve clinical outcome of AML patients.

Taken together, our data highlight the unique dual PIM and FLT3-ITD inhibitory activity of the SEL24-B489 that abrogates signaling circuits involved in proliferation, inhibition of apoptosis, protein translation/metabolism and supporting leukemia initiating cells. Given the favorable pharmacokinetics and safety profile, SEL24-B489 has been recently cleared for a phase I/II trial in AML patients. Thus, further clinical development of dual PIM/FLT3-ITD inhibition strategy with SEL24-B489 is ongoing.

## MATERIALS AND METHODS

### Chemicals

Dual PIM/FLT3-ITD inhibitor SEL24-B489 and PIM kinase inhibitor LGH447 were synthesized by Selvita. AZD1208 was purchased from ChemShuttle. Selective FLT3-ITD inhibitor AC220 and staurosporine were purchased from Selleckchem. Cytarabine (AraC) and vosaroxin were obtained from AK Scientific and Chem Scene, respectively. The compounds were prepared as 10mM stocks in DMSO (Sigma) and kept at −80°C until use. For *in vivo* studies, a hydrochloride salt of SEL24-B489 was used for its administration.

### Kinase assays

Dissociation constants (Kd) and inhibition levels of FLT3 mutants were measured at DiscoverX using the KINOME*scan*^TM^ Profiling Service (KdELECT).

### Cell lines and cell culture conditions

Cell lines were purchased from ATCC or DSMZ. MV-4-11 and SIG-M5 were cultured in IMDM, whereas MOLM-13, MOLM-14, MOLM-16, KG-1, Eol-1, SKM-1, PL-21, GDM-1, SKNO-1, RS4;11, Kasumi-3 and Kasumi-6 were cultured in RPMI medium, and OCI-AML-2 and OCI-AML-3 were cultured in Alpha-MEM medium. Media were supplemented with 10-20% FBS, 2 mM glutamine, 1 mM sodium pyruvate and 100 U/mL streptomycin/penicillin. In case of SKNO-1 and Kasumi-6 media were supplemented with 10 ng/ml and 2 ng/ml of GMC-SF, respectively. MOLM-14 cells expressing wild-type or mutant FLT3 receptors have been described previously [22]. Media and cell culture supplements were obtained from Lonza. Cell cultures were proven to be *Mycoplasma*-free using PCR technique to amplify the 16S rRNA region of *Mycoplasma* or using MycoAlert Mycoplasma Detection Kit (Lonza, Cat. No: LT07-318). All cell lines were incubated in a humidified atmosphere at 37 °C and 5% CO_2_.

### Viability assays and drug interactions

15-25 ×10^3^ of MV-4-11, MOLM-13, MOLM-14, MOLM-16, KG-1, Eol-1, SKM-1, PL-21, GDM-1, SKNO-1, RS4;11, Kasumi-3 and Kasumi-6 cells were seeded in 150 μL of culture medium onto individual wells in a 96 well plates, in triplicates. On the same day, 50 μL of medium containing tested inhibitor was added to the wells. Each compound was tested at 8 concentrations, in triplicates. As a negative control, the medium containing 0.1% DMSO was used. After 72 h incubation with the compounds, cell viability was assessed using the [3-(4,5-dimethylthiazol-2-yl)-5-(3-carboxymethoxyphenyl)-2-(4-sulfophenyl)-2H-tetrazolium (MTS) assay (Promega) and a microplate reader (Synergy 2, BioTek or Enspire). For viability assays of MOLM-14 cells expressing wild-type or mutant FLT3 receptors, 10^5^/mL cells were treated in triplicates with indicated concentrations of compounds. After 72 h, cell viability was determined in the MTS assay. To determine interactions between SEL24-B489 and AraC or a quinolone-derivative DNA-damaging agent, vosaroxin, MV-4-11 and MOLM-16 cells were seeded onto 96 well plates (5×10^3^ cells per well) in triplicates, and left for 18 h at 37°C, 5% CO_2_. Thereafter, diluted drugs were added to obtain a series of six increasing drug concentrations (final concentration of 0.003-0.325 μM for SEL24-B489, 0.036-3.525 μM for AraC, and 0.01-1.05 μM for vosaroxin). Staurosporine was used at 10 μM as a positive control. One the day of drug addition, basal signal from untreated cells was measured from in triplicate to determine time zero (T0). To determine interactions between AC220 and AZD1208, MV-4-11 and MOLM-16 cells were seeded onto 96 well plates (15×10^3^ cells per well) in triplicates. On the same day, diluted drugs were added in 3-fold dilutions (to obtain the final concentration 0.005-10 μM for AZD1208 and AC220 in MOLM-16 cells, while 0.005-10 μM for AZD1208 and 0.005-10 nM for AC220 in MV-4-11 cells). Cell viability was assessed after 72 h as described above. % of inhibition was determined relative to T0 as described in the Supplementary Document. GI50 and LC50 values were extrapolated from a variable (four-parameter) dose-response curve fitted in GraphPad Prism. The GI50 value corresponds to 50% for growth inhibition and the LC50 value corresponds to 50% death relative to T0. Combination indexes (CI) were determined using CompuSyn software (Paramus, NJ).

### Flow cytometry

For cell cycle analysis, MV4-11 and KG-1 cells were seeded at 1 × 10^6^ in 2 mL of their respective media. Cells were treated with the indicated compounds in 0.1% DMSO for 4 h, followed by a co-incubation with BrdU for 2 h (MV-4-11 and MOLM-13) or 3 h (KG-1). Cells were fixed for 20 min on ice in CytoFix/CytoPerm Solution (BD Biosciences) and further processed using the Apoptosis, DNA Damage and Cell Proliferation Kit (BD Biosciences) according to the manufacturer's instructions. Flow cytometry data were collected on the Attune NxT Flow Cytometer (Thermo Fisher) using filter settings for DAPI, PE PerCP-Cy5.5 and Alexa Fluor 647. Compensation was performed using the AbC™ Total Antibody Compensation Bead Kit (Thermo Fisher). Gating was applied to exclude debris and collect 200 000 singlet events. Data were analyzed and plotted in FlowJo (FlowJo, LCC) using autocompensation settings. Results are shown for approx. 50000 events.

For biomarker experiments, MV-4-11, MOLM-13 and KG-1 cells were seeded at 1 × 10^6^ in 3 ml of medium, while MOLM-16 cells were seeded at 1 × 10^6^ in 4 ml of medium and left for ~ 18 hours (37°C, 5% CO_2_). Thereafter, cells were treated with SEL24-B489 compound (0.01, 0.025, 0.1, 0.25, 0.5 1 μM). AC220 and LGH-447 were used at 1μM as positive control. Final concertation of DMSO was 0.1%. After 4h of incubation cells were harvested, centrifuged and washed with ice-cold PBS. Cells were fixed with 4% paraformaldehyde buffered with PBS and permeabilized with 90% methanol. Cells were incubated with primary antibody (S6 phopspho-Ser235/236, Cell Signaling, Cat. No: 4856) diluted 1:200 in 0.5% BSA/PBS. PE-conjugated secondary antibody (Anti-Rabbit PE-conjugated, Cell Signaling, Cat. No: 14705) was diluted 1:500 in 0.5% BSA/PBS. Cells were analyzed on Flow Cytometer BD LSR II with an excitation wavelength of 488 nm and peak emission 590 nm.

### Patient samples

Primary AML cells for *in vitro* analyses were obtained from peripheral blood of 5 newly diagnosed patients and one refractory to 3+7 daunorubicin and AraC induction treatment. All patients had peripheral leukemic blast counts exceeding 70% of total blood cell count at the time of sample collection. In addition, CD34+ blasts of 14 newly diagnosed AML patients were obtained with the CD34 MultiSort kit (Milteneyi Biotec) from cryopreserved bone marrow samples. Patient features are given in Supplementary Table 1. AML blasts were maintained in RPMI 1640 medium supplemented with 10% autologous plasma, 10% FBS (Lonza), 50 μg/ml streptomycin, 50 U/ml penicillin, 2 mM glutamine and 25mM HEPES buffer, at a density of 1 × 10^7^ cells/ml. Peripheral AML cells were incubated with indicated concentrations of AC220, AZD1208, SEL24-B489, or vehicle control for 48-96h. Bone marrow blasts were incubated with these compounds for 72h. Cell viability was determined using the propidium iodide (PI; BD Biosciences) exclusion assay in a FACS Canto flow cytometer (BD Biosciences).

### Immunoblotting

Cells were collected, washed twice with cold PBS, and lysed in RIPA buffer and kept on ice for 10 min. Tumor specimens from xenograft studies were homogenized in RIPA buffer using OMNI TH Tissue Homogenizer and kept on ice for 30 min. After incubation, cell and tumor lysates were centrifuged at 13,000×g at 4°C for 10 min and the supernatants were stored at −80°C until use. Protein concentration was measured using the Bradford method. For SDS-PAGE, 50 μg of protein in the Laemmli sample buffer was loaded onto 10% polyacrylamide gels and electrophoresed. Thereafter, proteins were transferred onto polyvinylidene fluoride (PVDF) membranes (Millipore). After blocking with 5% non-fat dry milk/TBS-T or 2% BSA/TBS-T, membranes were incubated with specific primary antibodies (listed in Supplementary Table 2) at 4 °C overnight. After extensive washing in PBS-T, membranes were incubated for 60 min with appropriate secondary peroxidase-conjugated IgG. The immunoreactive proteins were detected using an enhanced chemiluminescence (ECL) substrate (GE Healthcare) and visualized with Hyperfilm-ECL (GE Healthcare Biosciences).

### Animal studies

All animals were handled in strict accordance with good animal practice as defined by the relevant national and local animal welfare bodies. SCID and SCID/beige C.B-17 mice were provided by Harlan Laboratories or Charles River Laboratories, and were maintained in pathogen free conditions. For the xenograft experiments, age-matched (9-11 weeks old) females were used. 5×10^6^ of MV-4-11 or MOLM-16 cells were suspended in a mixture (1:1, v:v) of PBS with Matrigel (BD Biosciences,) and injected subcutaneously (sc), in a total volume of 100 μL, above the groin on the right hind limb. For MOLM-13 and KG-1 cells, a mixture (3:1, v:v) of PBS and Matrigel HC (BD Bioscience,) was used to inject 5 × 10^6^ of cell per mouse subcutaneously (sc), in a total volume of 100 μL. When tumors reached 100-130 mm^3^, mice were randomized into uniform groups (6-9 mice per group) and subsequently administered the compound. Prior to use, SEL24-B489 was freshly dissolved in water and administered at a dose of 25 mg/kg twice daily (BID), or 50, 75 and 100 mg/kg daily (QD), or 50 mg/kg every other day (EOD) by oral gavage (PO) using cannula in a volume of 10 μL per 1 g of body weight. At the end of the experiment, mice were anesthetized with isofluran and blood samples for total cell counts and biochemistry were obtained by retro-orbital bleeding. To assess the *in vivo* efficacy of SEL24-B489, AraC, or their combination, a MV-4-11 xenograft was established. 5×10^6^ cells suspended in a mixture (3:1, v:v) of PBS and Matrigel were injected as described above. When the tumor volume reached ~120 mm^3^, mice were randomized into uniform groups (5 mice per group) and the compound then administered. SEL24-B489 was administered by oral gavage (PO, 25 or 50 mg/kg, BID), and AraC was administered intraperitoneally (IP; 50 mg/kg, three times a week, TIW); each compound in a volume of 10 μL per 1 g of body weight. Tumor volume was monitored every second day and calculated using the formula: TV = (a^2^×b)/2, where “a” is the short axis in millimeters, and “b” is the long axis in millimeters. Body weight was assessed every day.

To evaluate SEL24-B489 pharmacodynamics (PD), MV-4-11 and MOLM-16 xenografts were established as described above. When tumor volume reached ~200 mm^3^, mice were randomized into uniform groups and administered SEL24-B489 by oral gavage (25 mg/kg BID for three consecutive days). At the end of the experiment, mice were sacrificed 4, 8 and 24h after the last dose (three mice per time point). Plasma and tumor samples were collected and stored at −80°C for further analysis.

### Statistical analyses

Differences in tumor growth kinetics were determined as indicated by factorial analysis of variance (ANOVA) with Tukey's post-hoc test, using SPSS v.17.0 software. Statistical differences in proliferation of MOLM-16 cells expressing FLT3 mutants and treated with SEL24-B489, AZD1208 or AC220 were determined by factorial ANOVA. P values < 0.05 were considered significant. Error bars represent standard deviations (SD) or standard error (SEM), indicated as appropriate.

## SUPPLEMENTARY MATERIALS FIGURES AND TABLES



## References

[1] Smith ML, Hills RK, Grimwade D (2011). Independent prognostic variables in acute myeloid leukaemia. Blood Rev.

[2] Rowe JM, Tallman MS (2010). How I treat acute myeloid leukemia. Blood.

[3] Döhner K, Döhner H (2008). Molecular characterization of acute myeloid leukemia. Haematologica.

[4] Gotze KS, Ramirez M, Tabor K, Small D, Matthews W, Civin CI (1998). Flt3high and Flt3low CD34+ progenitor cells isolated from human bone marrow are functionally distinct. Blood.

[5] Adolfsson J, Borge OJ, Bryder D, Theilgaard-Mönch K, Astrand-Grundström I, Sitnicka E, Sasaki Y, Jacobsen SE (2001). Upregulation of Flt3 expression within the bone marrow Lin(-)Sca1(+)c-kit(+) stem cell compartment is accompanied by loss of self-renewal capacity. Immunity.

[6] Thiede C, Steudel C, Mohr B, Schaich M, Schäkel U, Platzbecker U, Wermke M, Bornhäuser M, Ritter M, Neubauer A, Ehninger G, Illmer T (2002). Analysis of FLT3-activating mutations in 979 patients with acute myelogenous leukemia: association with FAB subtypes and identification of subgroups with poor prognosis. Blood.

[7] Levis M (2013). FLT3 mutations in acute myeloid leukemia: what is the best approach in 2013?. Hematology Am Soc Hematol Educ Program.

[8] Nakao M, Yokota S, Iwai T, Kaneko H, Horiike S, Kashima K, Sonoda Y, Fujimoto T, Misawa S (1996). Internal tandem duplication of the flt3 gene found in acute myeloid leukemia. Leukemia.

[9] Mizuki M, Schwable J, Steur C, Choudhary C, Agrawal S, Sargin B, Steffen B, Matsumura I, Kanakura Y, Bohmer FD, Muller-Tidow C, Berdel WE, Serve H (2003). Suppression of myeloid transcription factors and induction of STAT response genes by AML-specific Flt3 mutations. Blood.

[10] Janke H, Pastore F, Schumacher D, Herold T, Hopfner KP, Schneider S, Berdel WE, Büchner T, Woermann BJ, Subklewe M, Bohlander SK, Hiddemann W, Spiekermann K (2014). Activating FLT3 mutants show distinct gain-of-function phenotypes in vitro and a characteristic signaling pathway profile associated with prognosis in acute myeloid leukemia. PLoS One.

[11] Kim KT, Baird K, Ahn JY, Meltzer P, Lilly M, Levis M, Small D (2005). Pim-1 is up-regulated by constitutively activated FLT3 and plays a role in FLT3-mediated cell survival. Blood.

[12] Spiekermann K, Bagrintseva K, Schwab R, Schmieja K, Hiddemann W (2003). Overexpression and constitutive activation of FLT3 induces STAT5 activation in primary acute myeloid leukemia blast cells. Clin Cancer Res.

[13] Choudhary C, Brandts C, Schwable J, Tickenbrock L, Sargin B, Ueker A, Bohmer FD, Berdel WE, Muller-Tidow C, Serve H (2007). Activation mechanisms of STAT5 by oncogenic Flt3-ITD. Blood.

[14] Yanada M, Matsuo K, Suzuki T, Kiyoi H, Naoe T (2005). Prognostic significance of FLT3 internal tandem duplication and tyrosine kinase domain mutations for acute myeloid leukemia: a meta-analysis. Leukemia.

[15] Brault L, Gasser C, Bracher F, Huber K, Knapp S, Schwaller J (2010). PIM serine/threonine kinases in the pathogenesis and therapy of hematologic malignancies and solid cancers. Haematologica.

[16] Whitman SP, Archer KJ, Feng L, Baldus C, Becknell B, Carlson BD, Carroll AJ, Mrózek K, Vardiman JW, George SL, Kolitz JE, Larson RA, Bloomfield CD (2001). Absence of the wild-type allele predicts poor prognosis in adult de novo acute myeloid leukemia with normal cytogenetics and the internal tandem duplication of FLT3: a cancer and leukemia group B study. Cancer Res.

[17] Knapper S (2011). The clinical development of FLT3 inhibitors in acute myeloid leukemia. Expert Opin Investig Drugs.

[18] Moore AS, Faisal A, Gonzalez de Castro D, Bavetsias V, Sun C, Atrash B, Valenti M, de Haven Brandon A, Avery S, Mair D, Mirabella F, Swansbury J, Pearson AD (2012). Selective FLT3 inhibition of FLT3-ITD+ acute myeloid leukaemia resulting in secondary D835Y mutation: a model for emerging clinical resistance patterns. Leukemia.

[19] Smith CC, Wang Q, Chin CS, Salerno S, Damon LE, Levis MJ, Perl AE, Travers KJ, Wang S, Hunt JP, Zarrinkar PP, Schadt EE, Kasarskis A (2012). Validation of ITD mutations in FLT3 as a therapeutic target in human acute myeloid leukaemia. Nature.

[20] Williams AB, Nguyen B, Li L, Brown P, Levis M, Leahy D, Small D (2013). Mutations of FLT3/ITD confer resistance to multiple tyrosine kinase inhibitors. Leukemia.

[21] Alvarado Y, Kantarjian HM, Luthra R, Ravandi F, Borthakur G, Garcia-Manero G, Konopleva M, Estrov Z, Andreeff M, Cortes JE (2014). Treatment with FLT3 inhibitor in patients with FLT3-mutated acute myeloid leukemia is associated with development of secondary FLT3-tyrosine kinase domain mutations. Cancer.

[22] Green AS, Maciel TT, Hospital MA, Yin C, Mazed F, Townsend EC, Pilorge S, Lambert M, Paubelle E, Jacquel A, Zylbersztejn F, Decroocq J, Poulain L (2015). Pim kinases modulate resistance to FLT3 tyrosine kinase inhibitors in FLT3-ITD acute myeloid leukemia. Sci Adv.

[23] Fathi AT, Arowojolu O, Swinnen I, Sato T, Rajkhowa T, Small D, Marmsater F, Robinson JE, Gross SD, Martinson M, Allen S, Kallan NC, Levis M (2012). A potential therapeutic target for FLT3-ITD AML: PIM1 kinase. Leuk Res.

[24] Nawijn MC, Alendar A, Berns A (2011). For better or for worse: the role of Pim oncogenes in tumorigenesis. Nat Rev Cancer.

[25] Chen LS, Redkar S, Taverna P, Cortes JE, Gandhi V (2011). Mechanisms of cytotoxicity to Pim kinase inhibitor, SGI-1776, in acute myeloid leukemia. Blood.

[26] Klejman A, Schreiner SJ, Nieborowska-Skorska M, Slupianek A, Wilson M, Smithgall TE, Skorski T (2002). The Src family kinase Hck couples BCR/ABL to STAT5 activation in myeloid leukemia cells. EMBO J.

[27] Asano J, Nakano A, Oda A, Amou H, Hiasa M, Takeuchi K, Miki H, Nakamura S, Harada T, Fujii S, Kagawa K, Endo I, Yata K (2011). The serine/threonine kinase Pim-2 is a novel anti-apoptotic mediator in myeloma cells. Leukemia.

[28] Forshell LP, Li Y, Forshell TZ, Rudelius M, Nilsson L, Keller U, Nilsson J (2011). The direct Myc target Pim3 cooperates with other Pim kinases in supporting viability of Myc-induced B-cell lymphomas. Oncotarget.

[29] Szydłowski M, Prochorec-Sobieszek M, Szumera-Ciećkiewicz A, Derezińska E, Hoser G, Wasilewska D, Szymańska-Giemza O, Jabłońska E, Białopiotrowicz E, Sewastianik T, Polak A, Czardybon W, Gałęzowski M (2017). Expression of PIM kinases in Reed-Sternberg cells fosters immune privilege and tumor cell survival in Hodgkin lymphoma. Blood.

[30] Adam M, Pogacic V, Bendit M, Chappuis R, Nawijn MC, Duyster J, Fox CJ, Thompson CB, Cools J, Schwaller J (2006). Targeting PIM kinases impairs survival of hematopoietic cells transformed by kinase inhibitor-sensitive and kinase inhibitor-resistant forms of Fms-like tyrosine kinase 3 and BCR/ABL. Cancer Res.

[31] Agrawal S, Koschmieder S, Baumer N, Reddy NG, Berdel WE, Muller-Tidow C, Serve H (2008). Pim2 complements Flt3 wild-type receptor in hematopoietic progenitor cell transformation. Leukemia.

[32] Grundler R, Brault L, Gasser C, Bullock AN, Dechow T, Woetzel S, Pogacic V, Villa A, Ehret S, Berridge G, Spoo A, Dierks C, Biondi A (2009). Dissection of PIM serine/threonine kinases in FLT3-ITD-induced leukemogenesis reveals PIM1 as regulator of CXCL12-CXCR4-mediated homing and migration. J Exp Med.

[33] Qian KC, Studts J, Wang L, Barringer K, Kronkaitis A, Peng C, Baptiste A, LaFrance R, Mische S, Farmer B (2005). Expression, purification, crystallization and preliminary crystallographic analysis of human Pim-1 kinase. Acta Crystallogr Sect F Struct Biol Cryst Commun.

[34] Mikkers H, Nawijn M, Allen J, Brouwers C, Verhoeven E, Jonkers J, Berns A (2004). Mice deficient for all PIM kinases display reduced body size and impaired responses to hematopoietic growth factors. Mol Cell Biol.

[35] Foulks JM, Carpenter KJ, Luo B, Xu Y, Senina A, Nix R, Chan A, Clifford A, Wilkes M, Vollmer D, Brenning B, Merx S, Lai S (2014). A small-molecule inhibitor of PIM kinases as a potential treatment for urothelial carcinomas. Neoplasia.

[36] Baron BW, Anastasi J, Hyjek EM, Bies J, Reddy PL, Dong J, Joseph L, Thirman MJ, Wroblewski K, Wolff L, Baron JM (2012). PIM1 gene cooperates with human BCL6 gene to promote the development of lymphomas. Proc Natl Acad Sci U S A.

[37] Yang Q, Chen LS, Neelapu SS, Miranda RN, Medeiros LJ, Gandhi V (2012). Transcription and translation are primary targets of Pim kinase inhibitor SGI-1776 in mantle cell lymphoma. Blood.

[38] Siendones E, Barbarroja N, Torres LA, Buendía P, Velasco F, Dorado G, Torres A, López-Pedrera C (2007). Inhibition of Flt3-activating mutations does not prevent constitutive activation of ERK/Akt/STAT pathways in some AML cells: a possible cause for the limited effectiveness of monotherapy with small-molecule inhibitors. Hematol Oncol.

[39] Czardybon W, Brzozka K, Galezowski M, Windak R, Milik M, Zawadzka M, Guzik P, Wincza E, Prokop M, Wiklik K, Sabiniarz A, Cholody WM, Chorvath R, Selvita SA (2014). Novel benzimidazole derivatives as kinase inhibitors. Organization WIP.

[40] Keeton EK, McEachern K, Dillman KS, Palakurthi S, Cao Y, Grondine MR, Kaur S, Wang S, Chen Y, Wu A, Shen M, Gibbons FD, Lamb ML (2014). AZD1208, a potent and selective pan-Pim kinase inhibitor, demonstrates efficacy in preclinical models of acute myeloid leukemia. Blood.

[41] Kim W, Youn H, Seong KM, Yang HJ, Yun YJ, Kwon T, Kim YH, Lee JY, Jin YW, Youn B (2011). PIM1-activated PRAS40 regulates radioresistance in non-small cell lung cancer cells through interplay with FOXO3a, 14-3-3 and protein phosphatases. Radiat Res.

[42] Zhang F, Beharry ZM, Harris TE, Lilly MB, Smith CD, Mahajan S, Kraft AS (2009). PIM1 protein kinase regulates PRAS40 phosphorylation and mTOR activity in FDCP1 cells. Cancer Biol Ther.

[43] Lu J, Zavorotinskaya T, Dai Y, Niu XH, Castillo J, Sim J, Yu J, Wang Y, Langowski JL, Holash J, Shannon K, Garcia PD (2013). Pim2 is required for maintaining multiple myeloma cell growth through modulating TSC2 phosphorylation. Blood.

[44] Baldwin PR, Kapoor S, Natarajan K, Trotta R, Tron A, Huszar D, Davila E, Perrotti D, Baer MR (2016). Concurrent Inhibition of Pim-1 and FLT3 Kinases in FLT3-ITD Acute Myeloid Leukemia Post-Translationally Downregulates the Anti-Apoptotic Protein Mcl-1 through Downregulation of the Mcl-1 Deubiquitinase USP9X. Blood.

[45] Chu SH, Heiser D, Li L, Kaplan I, Collector M, Huso D, Sharkis SJ, Civin C, Small D (2012). FLT3-ITD knockin impairs hematopoietic stem cell quiescence/homeostasis, leading to myeloproliferative neoplasm. Cell Stem Cell.

[46] Li L, Piloto O, Nguyen HB, Greenberg K, Takamiya K, Racke F, Huso D, Small D (2008). Knock-in of an internal tandem duplication mutation into murine FLT3 confers myeloproliferative disease in a mouse model. Blood.

[47] Chung KY, Morrone G, Schuringa JJ, Wong B, Dorn DC, Moore MA (2005). Enforced expression of an Flt3 internal tandem duplication in human CD34+ cells confers properties of self-renewal and enhanced erythropoiesis. Blood.

[48] Moore MA, Dorn DC, Schuringa JJ, Chung KY, Morrone G (2007). Constitutive activation of Flt3 and STAT5A enhances self-renewal and alters differentiation of hematopoietic stem cells. Exp Hematol.

[49] Tam WF, Hahnel PS, Schuler A, Lee BH, Okabe R, Zhu N, Pante SV, Raffel G, Mercher T, Wernig G, Bockamp E, Sasca D, Kreft A (2013). STAT5 is crucial to maintain leukemic stem cells in acute myelogenous leukemias induced by MOZ-TIF2. Cancer Res.

[50] Natarajan K, Xie Y, Burcu M, Linn DE, Qiu Y, Baer MR (2013). Pim-1 kinase phosphorylates and stabilizes 130 kDa FLT3 and promotes aberrant STAT5 signaling in acute myeloid leukemia with FLT3 internal tandem duplication. PLoS One.

[51] Sewastianik T, Prochorec-Sobieszek M, Chapuy B, Juszczynski P (2014). MYC deregulation in lymphoid tumors: molecular mechanisms, clinical consequences and therapeutic implications. Biochim Biophys Acta.

[52] Zhang Y, Wang Z, Li X, Magnuson NS (2008). Pim kinase-dependent inhibition of c-Myc degradation. Oncogene.

[53] Guo Z, Wang A, Zhang W, Levit M, Gao Q, Barberis C, Tabart M, Zhang J, Hoffmann D, Wiederschain D, Rocnik J, Sun F, Murtie J (2014). PIM inhibitors target CD25-positive AML cells through concomitant suppression of STAT5 activation and degradation of MYC oncogene. Blood.

[54] Tamburini J, Green AS, Bardet V, Chapuis N, Park S, Willems L, Uzunov M, Ifrah N, Dreyfus F, Lacombe C, Mayeux P, Bouscary D (2009). Protein synthesis is resistant to rapamycin and constitutes a promising therapeutic target in acute myeloid leukemia. Blood.

[55] Kim KT, Levis M, Small D (2006). Constitutively activated FLT3 phosphorylates BAD partially through pim-1. Br J Haematol.

[56] Harada M, Benito J, Yamamoto S, Kaur S, Arslan D, Ramirez S, Jacamo R, Platanias L, Matsushita H, Fujimura T, Kazuno S, Kojima K, Tabe Y (2015). The novel combination of dual mTOR inhibitor AZD2014 and pan-PIM inhibitor AZD1208 inhibits growth in acute myeloid leukemia via HSF pathway suppression. Oncotarget.

[57] Hu YL, Passegue E, Fong S, Largman C, Lawrence HJ (2007). Evidence that the Pim1 kinase gene is a direct target of HOXA9. Blood.

