# Supplementary material for: A novel, dual pan-PIM/FLT3 inhibitor SEL24 exhibits broad therapeutic potential in acute myeloid leukemia
Source: Oncotarget. 2018 Mar 30;9(24):16917–31. doi: 10.18632/oncotarget.24747 (PMC5908295; doi:10.18632/oncotarget.24747)
Supplement: Supplementary file 1 [file oncotarget-09-16917-s001.pdf]

A novel, dual pan-PIM/FLT3 inhibitor SEL24 exhibits broad therapeutic potential in acute myeloid leukemia

SUPPLEMENTARY MATERIALS

Efficacy calculations in MTS (viability assay)

MTS raw data values, after being blank-corrected to wells containing growth medium and DMSO only, are pasted into the pre-formatted Excel template spread sheet for further calculations. The absorbance values that are higher than average "time 0 plate" absorbance value, are calculated as % of control, vehicle-treated cells at 144h, according to the following formula:

$$\frac{\text{Compound treated cells Abs value} - \text{“time 0” average Abs value}}{\text{Control Abs value} - \text{“time 0” average Abs value}} *100$$

Calculated values are in the range of 0% - 100% and indicate the cytostatic effect of the compound. The absorbance values that are lower than average "time 0 plate" absorbance value, are calculated as % of average "time 0 plate" values, according to the following formula:

$$\frac{\text{Compound treated cells Abs value} - \text{“time 0” average Abs value}}{\text{“time 0” average Abs value}} *100$$

Calculated values are in the range of -100% to 0% and indicate the cytotoxic effect of the compound.

SEL24-B489 pharmacokinetics, metabolic stability and CYP inhibition

Pharmacokinetics properties of SEL24-B489 were analyzed in three different species. CD-1 mice (three females per timepoint) were dosed with 50 mg/kg by oral gavage (PO) and with 15 mg/kg given intravenously (IV). Three SD male rats were treated with 25 mg/kg PO or 1 mg/kg IV. Three male Beagle dogs were administered 5 mg/kg PO and 1 mg/kg IV. SEL24-B489 was freshly dissolved in water at the required concentrations. To assess pharmacokinetic properties of a compound, whole blood was collected at the selected timepoints, plasma was extracted and stored at -80 °C for further analyses. PK parameters of SEL24-B489 are given in Supplementary Table 6. Of note, the compound exhibited good oral bioavailability calculated as 62 % in mice, 52 % in rats and 47 % in dogs.

Stability of SEL24-B489 was tested in the Liver S9 fraction as well as in liver microsomes across all tested species – mouse, rat, dog, human. Tested compounds (at initial concentration of 1 µM) were incubated at 37 °C

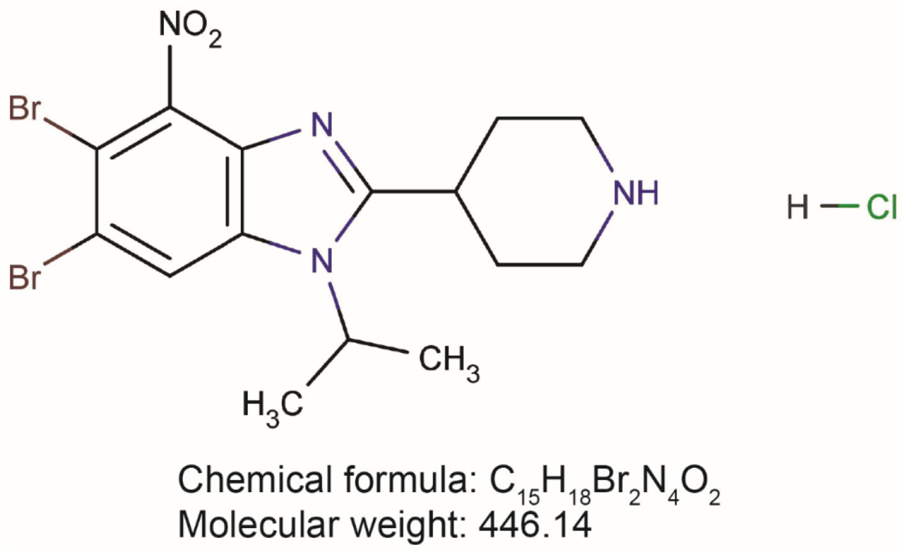

Supplementary Figure 1: Chemical structure of SEL24-B489. (5,6-dibromo-4-nitro-2-)piperidin-4-yl)-1-(propan-2-yl)-1H-1,3-benzimidazole). Chemical formula and molecular weight are given for free base.

with liver microsomes or the S9 fraction in the presence or absence of enzyme cofactors. The assay was performed in triplicate, at four time points (0, 20, 40 and 60 minutes) with the respective reference samples (i.e. without cofactors). After incubation, drug transformation by liver enzymes was terminated by adding cold acetonitrile. The loss of compound was determined by LC-MS analysis. Results were adjusted for reaction volume and protein concentration, and presented as intrinsic clearance. The analysis of two standard compounds (i.e. positive and negative controls) was included. In these analyses, SEL24-B489 exhibited good stability, with a half-life ($T_{1/2}$) varying from 2.6 hours in mice to 9.9 hours in the human S9 fraction, and 7.2 hours in mice to 21.1 hours in human microsomes. (Supplementary Table 7).

SEL24-B489 CYP-inhibitory activity was measured at Cambridge Bioscience Limited (except CYP1A2 inhibition which was measured in-house). The compound was tested in a 5-dose IC50 mode with 3-fold serial dilution starting at 30 µM. The control compound Ketoconazole was tested in a 10-dose IC50 with 3-fold serial dilution starting at 10 µM. SEL24-B489 showed a very weak potential for CYP inhibition with IC50 between 12.6 and 22.1 µM (Supplementary Table 8), indicating its low potential for causing drug-drug interaction.

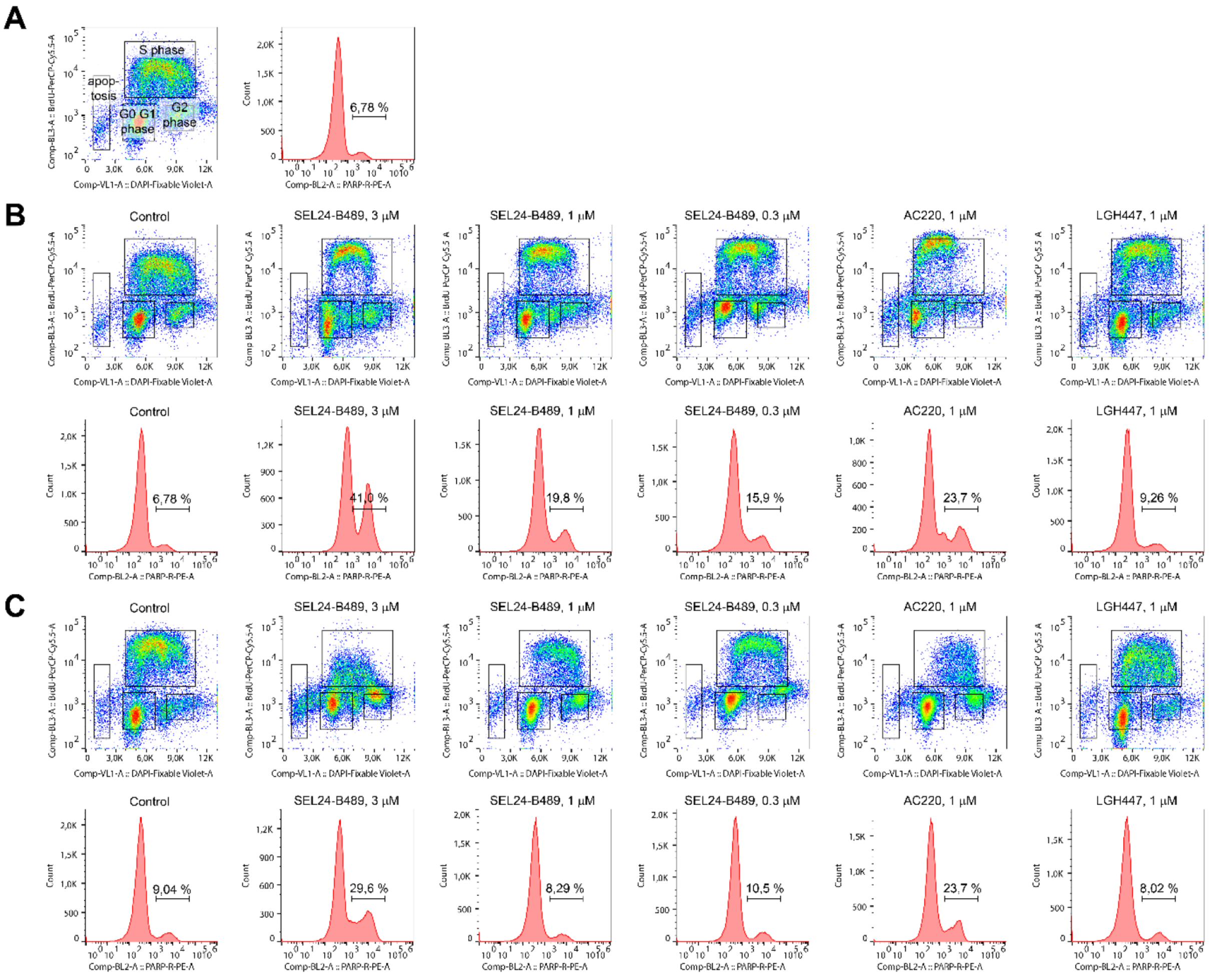

Supplementary Figure 2: Cell cycle and apoptosis analysis in MV-4-11 (FLT3-ITD+) cells treated with SEL24-B489, AC220 and LGH447. **(A)** Gating was applied to discriminate cells from debris. In the single cell population, cell cycle progression was analyzed based on the BrdU incorporation (Y axis) and DAPI signal to determine DNA content (X axis), as shown for control samples (left panel). Gating was applied, as demonstrated for control samples, to determine the percentage of single cells with pleaved PARP (shown in red). **(B)** MV-4-11 cells were treated as indicated for 4 hours. Upper panel: signal from the BrdU-PerCP-Cy5.5 antibody is plotted on the Y axis of dot plots and signal from DAPI is plotted on the X axis. Lower panel: signal for the PARP-PE antibody is shown on histogram graphs for cells treated as indicated. **(C)** MV-4-11 cells were treated as indicated for 24 hours. Upper panel: signal from the BrdU-PerCP-Cy5.5 antibody is plotted on the Y axis of dot plots and signal from DAPI is plotted on the X axis. Lower panel: signal for the PARP-PE antibody is shown on histogram graphs for cells treated as indicated.

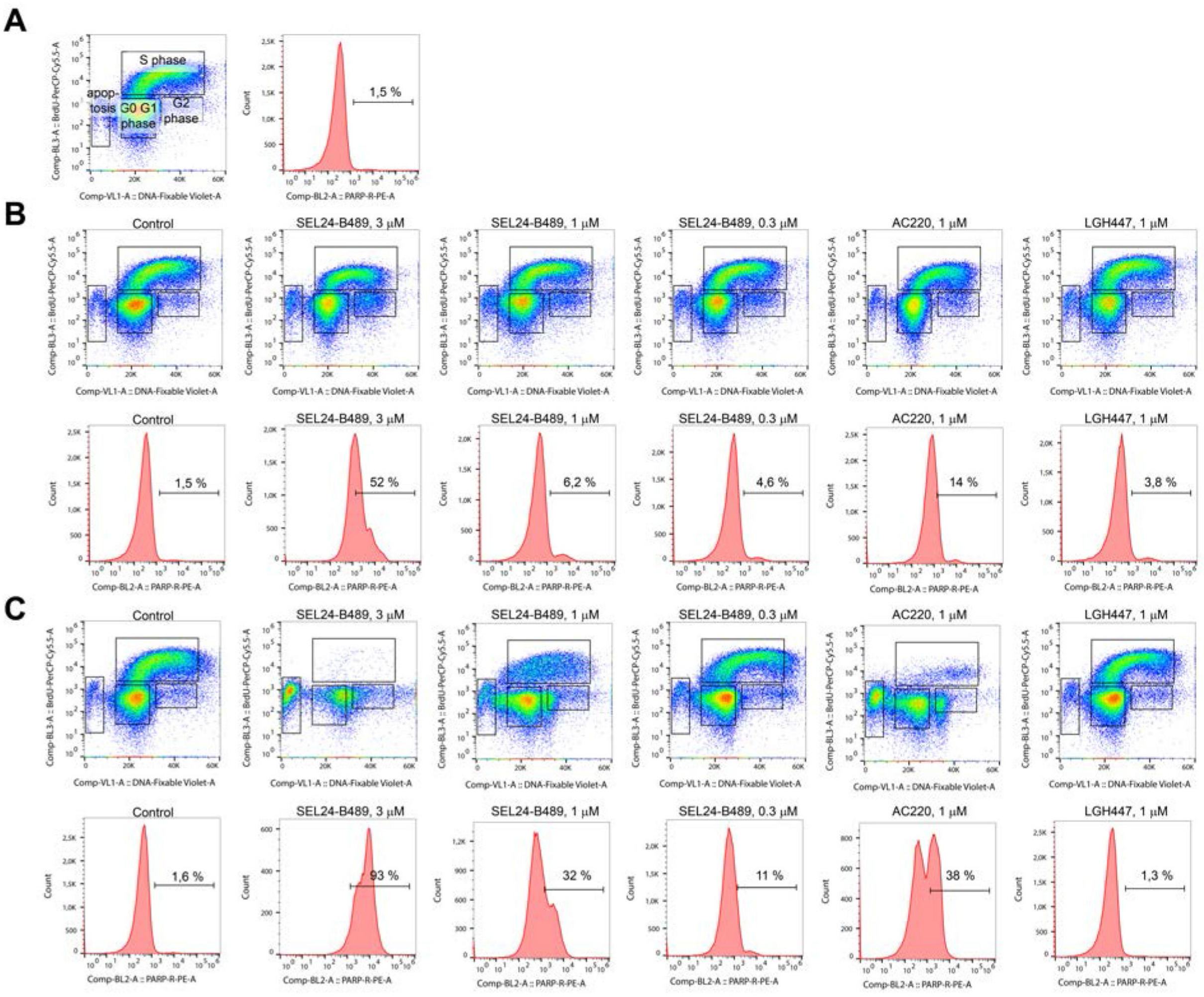

Supplementary Figure 3: Cell cycle and apoptosis analysis in MOLM-13 (FLT3-ITD+) cells treated with SEL24-B489, AC220 and LGH447. (A) Gating was applied to discriminate cells from debris. In the single cell population, cell cycle progression was analyzed based on the BrdU incorporation (Y axis) and DAPI signal to determine DNA content (X axis), as shown for control samples (left panel). Gating was applied, as demonstrated for control samples, to determine the percentage of single cells with pleaved PARP (shown in red). **(B)** MOLM-13 cells were treated as indicated for 4 hours. Upper panel: signal from the BrdU-PerCP-Cy5.5 antibody is plotted on the Y axis of dot plots and signal from DAPI is plotted on the X axis. Lower panel: signal for the PARP-PE antibody is shown on histogram graphs for cells treated as indicated. **(C)** MOLM-13 cells were treated as indicated for 24 hours. Upper panel: signal from the BrdU-PerCP-Cy5.5 antibody is plotted on the Y axis of dot plots and signal from DAPI is plotted on the X axis. Lower panel: signal for the PARP-PE antibody is shown on histogram graphs for cells treated as indicated.

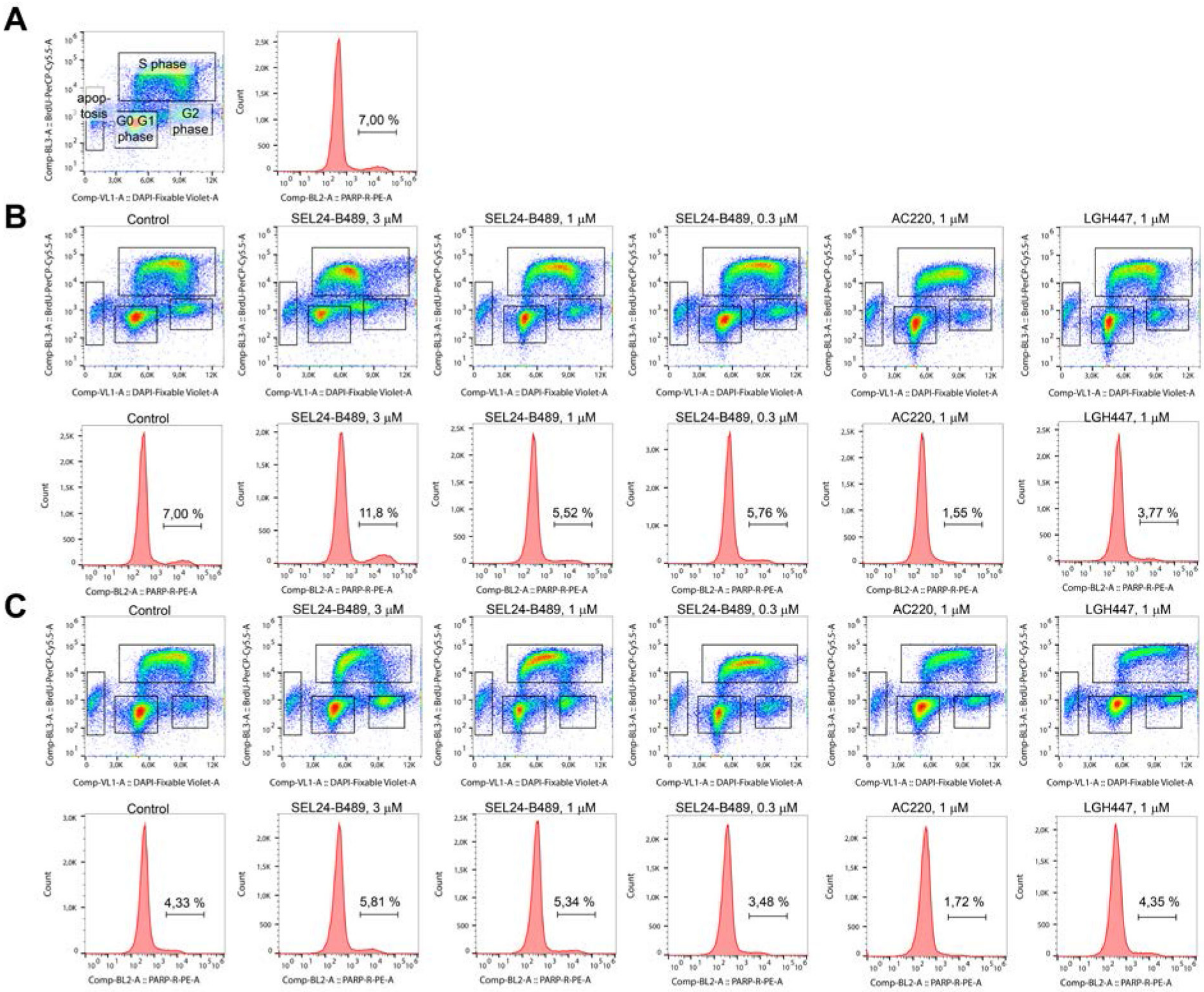

Supplementary Figure 4: Cell cycle and apoptosis analysis in KG-1 (FLT3-WT) cells treated with SEL24-B489, AC220 and LGH447. **(A)** Gating was applied to discriminate cells from debris. In the single cell population, cell cycle progression was analyzed based on the BrdU incorporation (Y axis) and DAPI signal to determine DNA content (X axis), as shown for control samples (left panel). Gating was applied, as demonstrated for control samples, to determine the percentage of single cells with pleaved PARP (shown in red). **(B)** KG-1 cells were treated as indicated for 4 hours. Upper panel: signal from the BrdU-PerCP-Cy5.5 antibody is plotted on the Y axis of dot plots and signal from DAPI is plotted on the X axis. Lower panel: signal for the PARP-PE antibody is shown on histogram graphs for cells treated as indicated. **(C)** KG-1 cells were treated as indicated for 24 hours. Upper panel: signal from the BrdU-PerCP-Cy5.5 antibody is plotted on the Y axis of dot plots and signal from DAPI is plotted on the X axis. Lower panel: signal for the PARP-PE antibody is shown on histogram graphs for cells treated as indicated.

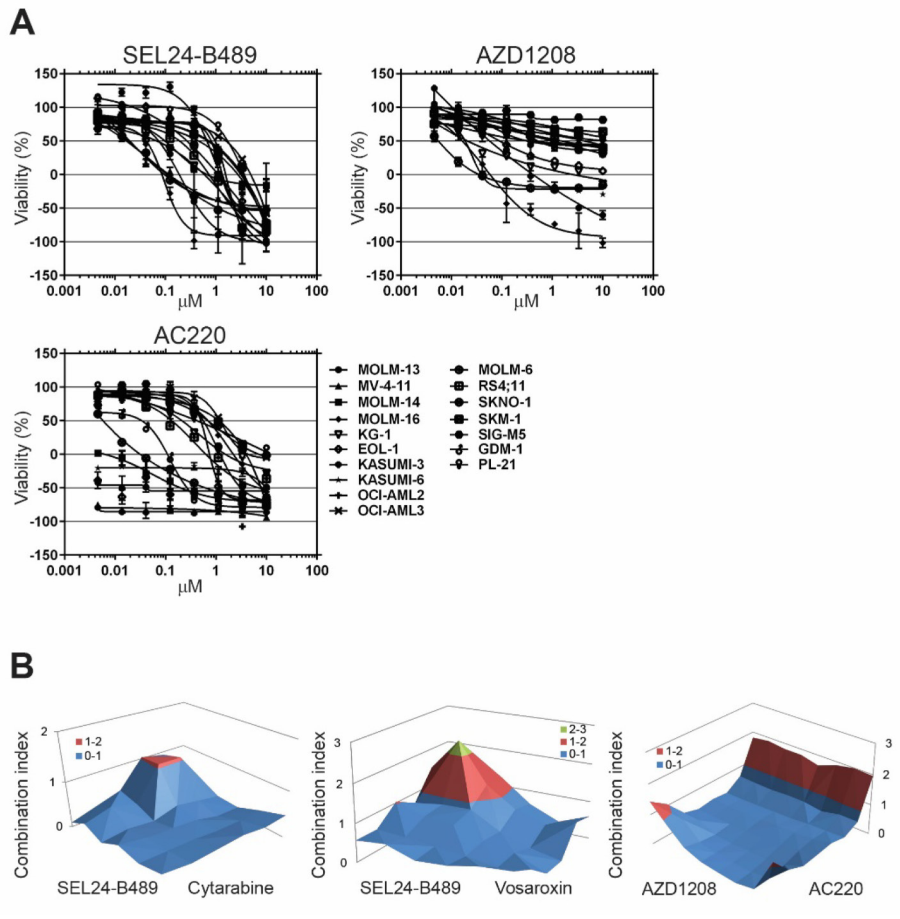

Supplementary Figure 5: *In vitro* activity of SEL24-B489 in AML cell lines. (A) *In vitro* activity of SEL24-B489, AZD1208 and AC220 in AML cell lines was assessed using an MTS assay. Cells viability was measured after 72 h incubation with three-fold serial dilutions of compounds, starting at 10 μM concentration. Obtained data were presented as percentage of viable cells compared with control (untreated) cells viability. Error bars indicate SD. **(B)** Pharmacological interactions between SEL24-B489 with AraC or vosaroxin in MV-4-11 cells. Combination index (CI) are shown on the Y axis and compound concentrations are shown on X and Z axes. Values below 1 indicate an additive effect and below 0.7- a synergy between tested compounds. Values above 1 indicate an antagonistic effect.

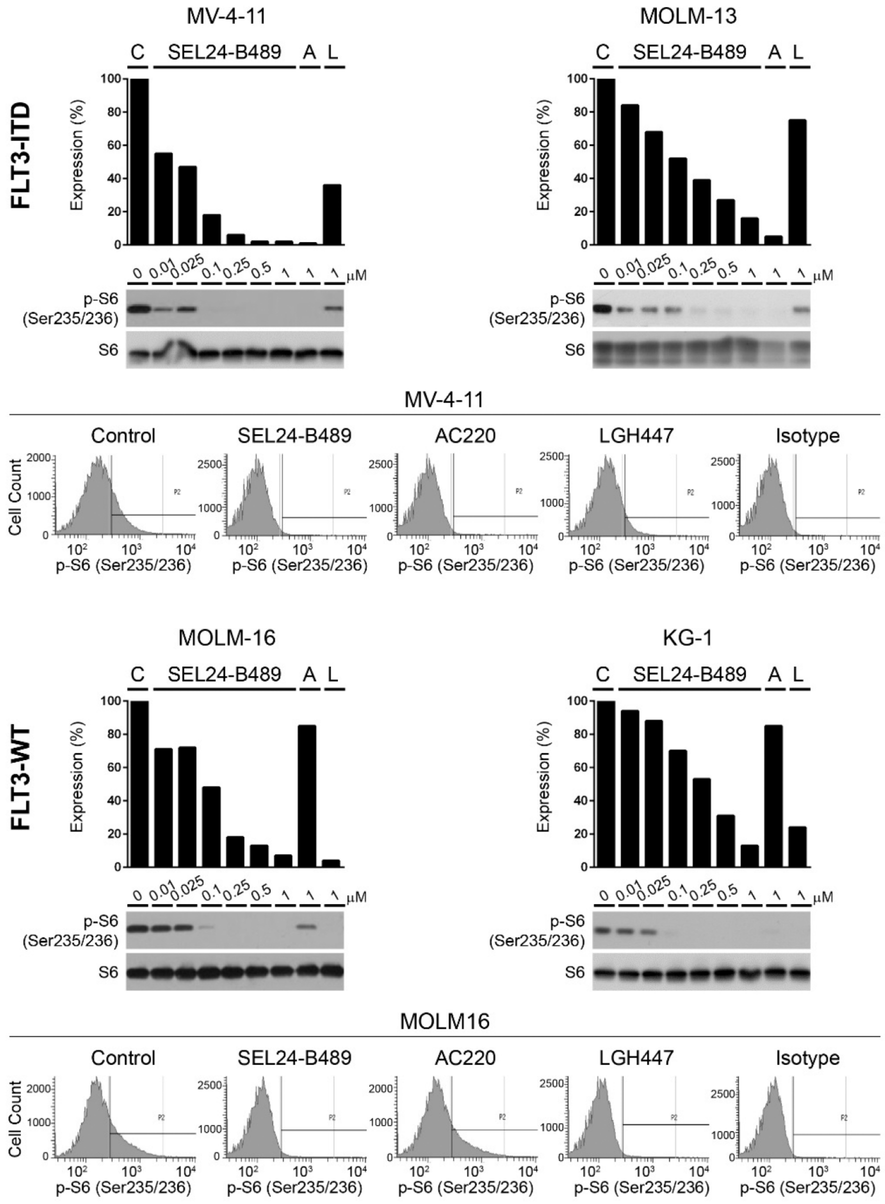

Supplementary Figure 6: Analysis of S6 phosphorylation (Ser235/236) levels in AML cell lines. MV-4-11 (FLT3-ITD+), MOLM-13 (FLT3-ITD+), MOLM-16 (FLT3-WT) and KG-1 (FLT3-WT) cells expressing high levels of PIM kinases, were treated with SEL24-B489 for 4 hours in a dose-dependent manner and analyzed for phosphorylation of S6 kinase using flow cytometer (charts) and Western blot (photographs). A – AC220 and L – LGH-447 compounds were used as reference. C – vehicle control.

Supplementary Table 1: Clinical characteristics of patients in the study

No	Age	Sex	FAB	Status	Mutation								Karyotype
					AML1-ETO; RUNX1-RUNX1T1	CBFB-MYH11	MLL-PTD	FLT3-ITD	PML-RARA	BAALC over-expression	CEBPA mutation	NPM1 mutation	
**1**	62	M	M2	RR	-	-	-	-	NA	NA	NA	NA	Complex
**2**	53	M	M2	N	-	-	-	-	-	+	NA	NA	46, XY
**3**	44	F	M7	N	-	-	-	+	-	+	-	+	Metaphases not obtained
**4**	59	F	U	N	-	-	-	+	-	-	-	+	46, XX
**5**	34	M	M1	N	-	-	+	-	-	+	-	-	46, XY
**6**	56	F	M2	N	-	-	-	+	-	+	NA	NA	46, XX, del(16)(q12q22)[6]
**7**	84	M	M1/M2	N	NA	NA	NA	NA	NA	NA	NA	NA	Complex
**8**	33	F	M2	N	-	-	-	-	NA	+	NA	-	46, XX
**9**	59	F	M2	N	-	-	-	-	NA	+	NA	NA	46, XX
**10**	75	M	M2	N	NA	NA	NA	+	NA	NA	NA	NA	46, XY
**11**	63	M	M2	N	-	-	-	-	NA	-	-	-	46, XY
**12**	40	F	M2	N	-	-	-	-	NA	+	NA	-	46, XX, inv(16) + tri(8)
**13**	63	F	M2	N	-	-	-	-	NA	+	-	NA	46, XX, del(5)(q32q34), del(12)(p11.2)
**14**	65	F	M2	N	-	-	-	-	NA	+	-	NA	46, XX, del(7)(q22q36)
**15**	59	M	RAEB-2	N	-	-	-	-	NA	+	NA	NA	Complex
**16**	63	M	M2	N	-	-	-	-	NA	+	NA	NA	45, X0, del(12)(p11.2)
**17**	75	F	M4	N	-	-	-	+	NA	-	+	+	46, XX
**18**	70	F	M2	N	NA	NA	-	-	NA	NA	NA	NA	45, XX, -7
**19**	72	M	M2	N	NA	NA	-	-	NA	+	NA	NA	46, XY

Samples no 1-6 - peripheral blood; Samples no 7-19 - bone marrow; N- newly diagnosed, previously untreated; RR – relapsed or refractory, previously treated; NA – not assessed; U-unclassifiable.

Supplementary Table 2: List of antibodies used in the study

Antibody name	Producer	Catalog number	LOT number
**cMYC (phospho-Ser62)**	Assay bioTech	A0020	102612110020
**cMYC**	Abcam	ab32072	GR85451-4
**4EBP1 (phospho-Ser65)**	Cell Signaling	9456	5
**4EBP1 (phospho-Thr37/46)**	Cell Signaling	2855	17
**4EBP1**	Abcam	ab2606	GR19075-2
**S6 (phospho-Ser235/236)**	Abcam	ab80158	960019
**S6 (phospho-Ser240/244)**	Cell Signaling	5364	3
**S6 ribosomal**	Cell Signaling	2217	5
**MCL1**	Abcam	ab32087	GR42511-2
**PARP**	Cell Signaling	9542	11
**S6K (phospho-Thr389)**	Cell Signaling	8209	7
**S6K**	Cell Signaling	2708	4
**ERK1/2 (phospho-Thr202/ Tyr204)**	Cell Signaling	4370	9
**ERK1/2**	Cell Signaling	4695	14
**STAT5 (phospho-Ser726)**	Abcam	ab128896	GR97838-5
**STAT5**	Santa Cruz	sc-835	G1212
**Tubulin**	Sigma	T5192	109K4802

Supplementary Table 3: *In vitro* activity of SEL24-B489 in AML cell lines – GI50. Cell viability after 72 h incubation with serial dilutions of SEL24-B489, AZD-1208, AC220 and cytarabine, was determined using the MTS assay. GI50 (50% of growth inhibition) was determined from the sigmoidal dose-response curve (GraphPad Prism). FLT3-ITD+ cell lines: MV-4-11, MOLM-13. FLT3-WT cell lines: MOLM-14, MOLM-16, KG-1, EOL-1, KASUMI-3, KASUMI-6, OCI-AML-2, OCI-AML-3, MOLM-6, RS4;11, SKNO-1, SKM-1, SIG-M5, GDM-1, PL-21.

	SEL24-B489	AZD1208	AC220	Cytarabine
**MV-4-11**	0.020	1.463	<0.005	0.340
**MOLM-13**	0.073	0.306	<0.005	<0.005
**MOLM-14**	0.097	-	<0.005	0.048
**MOLM-16**	0.042	0.016	0.688	0.022
**KG-1**	0.067	0.020	0.210	0.012
**EOL-1**	0.266	0.089	<0.005	0.024
**KASUMI-3**	0.633	0.057	<0.005	<0.005
**KASUMI-6**	0.017	0.019	<0.005	<0.005
**OCI-AML-2**	0.960	0.333	0.499	<0.005
**OCI-AML-3**	1.588	>10	1.185	0.081
**MOLM-6**	0.449	0.973	0.681	0.030
**RS4;11**	0.190	4.221	0.132	<0.005
**SKNO-1**	0.018	0.005	0.006	<0.005
**SKM-1**	0.732	>10	1.131	0.028
**SIG-M5**	0.806	>10	0.558	0.046
**GDM-1**	0.680	0.506	0.033	<0.005
**PL-21**	1.709	>10	0.412	<0.005

Supplementary Table 4: *In vitro* activity of SEL24-B489 in AML cell lines – LC50. Cell viability after incubation with indicated drugs was assessed as in Supplementary Table 3 . The lethal concentration was assessed and LC50 (50% of lethal concentration) was determined from the sigmoidal dose-response curve (GraphPad Prism). FLT3-ITD+ cell lines: MV-4-11, MOLM-13. FLT3-WT cell lines: MOLM-14, MOLM-16, KG-1, EOL-1, KASUMI-3, KASUMI-6, OCI-AML-2, OCI-AML-3, MOLM-6, RS4;11, SKNO-1, SKM-1, SIG-M5, GDM-1, PL-21.

	SEL24-B489	AZD1208	AC220	Cytarabine
**MV-4-11**	>10	>10	<0.005	>10
**MOLM-13**	0.446	>10	0.042	>10
**MOLM-14**	>10	-	0.196	>10
**MOLM-16**	0.153	0.233	8.229	>10
**KG-1**	2.441	>10	>10	0.071
**EOL-1**	3.892	>10	<0.005	6.195
**KASUMI-3**	2.33	4.633	<0.005	0.028
**KASUMI-6**	4.279	>10	9.937	>10
**OCI-AML-2**	2.324	>10	1.239	0.021
**OCI-AML-3**	9.206	>10	>10	>10
**MOLM-6**	7.205	>10	>10	>10
**RS4;11**	2.156	>10	6.956	0.023
**SKNO-1**	1.094	>10	0.612	0.025
**SKM-1**	7.772	>10	7.794	6.456
**SIG-M5**	3.444	>10	3.094	>10
**GDM-1**	7.061	>10	0.266	0.478
**PL-21**	7.051	>10	>10	>10

Supplementary Table 5: Analysis of S6 phosphorylation (Ser235/236) levels in AML cell lines. MV-4-11 (FLT3-ITD+), MOLM-13 (FLT3-ITD+), MOLM-16 (FLT3-WT) and KG-1 (FLT3-WT) cells expressing high levels of PIM kinases, were treated with SEL24-B489 for 4 hours in a dose-dependent manner and analyzed for phosphorylation of S6 kinase using flow cytometer and Western blot. IC50 (inhibitory concentration) was calculated for both flow cytometry and Western blot analyses.

	IC50 (µM)	
	**Flow cytometry**	**Western blotting**
**MV-4-11**	0.013	0.001
**MOLM-13**	0.102	0.003
**MOLM-16**	0.058	0.035
**KG-1**	0.195	0.031

Supplementary Table 6: Pharmacokinetic properties of SEL24-B489 in mice, rats and dogs. Pharmacokinetic profiles of SEL24-B489 in mice (CD-1), rats (Sprague Dawley) and dogs (Beagle) were determined after oral and intravenous administration. PO – per os, IV – intravenous, $T_{1/2}$ – elimination half-life, C_{max} – peak plasma concentration, T_{max} – time to reach C_{max}, AUC – area under the curve, F – bioavailability.

	Dose PO/IV [mg/kg]	$T_{1/2}$ [h]	C_{max} [ng/mL]	T_{max} [h]	AUC_{0-t} [h*ng/mL]	F [%]
**Mouse**	50/15	5.2	2185	4	26176	62
**Rat**	25/1	10	913	6.7	19630	52
**Dog**	5/1	17.6	155	1.75	3078	47

Supplementary Table 7: Metabolic stability of SEL24-B489. SEL24-B489 was incubated with the liver S9 fraction or with liver microsomes. Compound's half-life time was measured with LC-MS.

	Liver S9 fraction $t_{1/2}$[h]	Liver microsomes $t_{1/2}$[h]
**Mouse**	2.6	7.2
**Rat**	4.3	21.8
**Dog**	6.3	7.6
**Human**	9.9	21.1

Supplementary Table 8: CYP isoforms inhibition. The compound was tested in a 5-dose IC50 mode with 3-fold serial dilution starting at 30 [µM]. CYP activity was assessed as described in the Supplementary methods as above.

	IC50 [µM]						
	**CYP1A2**	**CYP3A4**	**CYP2B6**	**CYP2C8**	**CYP2C9**	**CYP2C19**	**CYP2D6**
**SEL24-B489**	>30	>100	>40	>30	>150	>70	10

Supplementary Table 9: Related to Figure 5C. Safety of SEL24-B489 in AML xenograft model. The assessment of blood biochemistry in mice with MV-4-11 tumors treated with SEL24-B489 in combination with AraC (as in Figure 5C) was performed on treatment day 15.

	AST U/L	ALT U/L	ALP U/L	UREA mmol/L	CREA mg/dL	CK U/L	TP g/dL	ALB g/dL	Na nmol/L	K nmol/L
**Control**	161.0	56.5	81.8	5.0	0.45	974	4.3	3.6	143.3	5.0
**SEL24-B489, 50 mg/kg**	135.2	49.4	97.2	6.6	0.49	1206	5.1	4.4	141.2	5.3
**SEL24-B489, 25 mg/kg**	202.4	70.5	94.2	6.0	0.40	2076	5.6	5.3	138.2	5.3
**Cytarabine, 50 mg/kg**	247.1	153.7	87.6	5.9	0.37	1709	4.6	4.6	138.4	5.4
**SEL24-B489A, 50 mg/kg + Cytarabine, 50 mg/kg**	121.4	70.5	103.2	7.4	0.39	1168	5.0	5.0	135.0	5.4
**SEL24-B489A, 25 mg/kg + Cytarabine, 50 mg/kg**	210.6	69.0	79.2	6.4	0.30	1859	4.3	4.1	137.6	5.2

AST – Aspartate aminotransferase, ALT – Alanine transaminase, ALP – alkaline phosphatase, UREA – blood urea nitrogen, CREA – Creatinine, CK – Creatine kinase, TP- total protein; ALB – Albumin.

Supplementary Table 10: Related to Figure 5C. Safety of SEL24-B489 in AML xenograft model. The assessment total blood cell counts in mice with MV-4-11 tumors treated with SEL24-B489 in combination with AraC (as in Figure 5C) was performed on treatment day 15.

	RBC $10^6/mm^3$	HGB g/dL	HCT %	WBC $10^3/mm^3$	LYMPH %	MONO %	GRA %	PLT $10^3/mm^3$	MPV μm^3
**Control**	9.6	14.2	48.5	4.8	36.6	10.6	52.8	1091	5.9
**SEL24-B489, 50 mg/kg**	9.6	14.4	47.4	4.9	40.3	10.5	49.1	1286	5.7
**SEL24-B489, 25 mg/kg**	9.8	14.6	48.8	3.1	47.2	10.6	42.2	1016	6.1
**Cytarabine, 50 mg/kg**	9.9	14.4	49.5	5.0	37.7	10.3	51.9	1098	5.9
**SEL24-B489A, 50 mg/kg + Cytarabine, 50 mg/kg**	8.8	13.2	43.7	4.3	36.4	11.9	51.7	1196	5.5
**SEL24-B489A, 25 mg/kg + Cytarabine, 50 mg/kg**	9.8	14.7	48.5	5.5	37.2	12.9	49.9	1313	5.7

RBC – red blood cells, HGB – hemoglobin, HCT – hematocrit, WBC – white blood cells, LYMPH – lymphocytes, MONO – monocytes, GRA – granulocytes, PLT – platelet, MPV – mean platelet volume.
